# Interrogating two extensively self-targeting Type I CRISPR-Cas systems in *Xanthomonas albilineans* reveals distinct anti-CRISPR proteins that block DNA degradation

**DOI:** 10.1093/nar/gkad1097

**Published:** 2023-11-28

**Authors:** Franziska Wimmer, Frank Englert, Katharina G Wandera, Omer S Alkhnbashi, Scott P Collins, Rolf Backofen, Chase L Beisel

**Affiliations:** Helmholtz Institute for RNA-based Infection Research (HIRI), Helmholtz Centre for Infection Research (HZI), 97080 Würzburg, Germany; Helmholtz Institute for RNA-based Infection Research (HIRI), Helmholtz Centre for Infection Research (HZI), 97080 Würzburg, Germany; Helmholtz Institute for RNA-based Infection Research (HIRI), Helmholtz Centre for Infection Research (HZI), 97080 Würzburg, Germany; Information and Computer Science Department, King Fahd University of Petroleum and Minerals (KFUPM), Dhahran 31261, Saudi Arabia; Interdisciplinary Research Center for Intelligent Secure Systems (IRC-ISS), King Fahd University of Petroleum and Minerals (KFUPM), Dhahran 31261, Saudi Arabia; Department of Chemical & Biomolecular Engineering, North Carolina State University, Raleigh, NC 27695, USA; Bioinformatics group, Department of Computer Science, University of Freiburg, Freiburg, Germany; Signalling Research Centres BIOSS and CIBSS, University of Freiburg, Freiburg, Germany; Helmholtz Institute for RNA-based Infection Research (HIRI), Helmholtz Centre for Infection Research (HZI), 97080 Würzburg, Germany; Department of Chemical & Biomolecular Engineering, North Carolina State University, Raleigh, NC 27695, USA; Medical Faculty, University of Würzburg, 97080 Würzburg, Germany

## Abstract

CRISPR-Cas systems store fragments of invader DNA as spacers to recognize and clear those same invaders in the future. Spacers can also be acquired from the host's genomic DNA, leading to lethal self-targeting. While self-targeting can be circumvented through different mechanisms, natural examples remain poorly explored. Here, we investigate extensive self-targeting by two CRISPR-Cas systems encoding 24 self-targeting spacers in the plant pathogen *Xanthomonas albilineans*. We show that the native I-C and I-F1 systems are actively expressed and that CRISPR RNAs are properly processed. When expressed in *Escherichia coli*, each Cascade complex binds its PAM-flanked DNA target to block transcription, while the addition of Cas3 paired with genome targeting induces cell killing. While exploring how *X. albilineans* survives self-targeting, we predicted putative anti-CRISPR proteins (Acrs) encoded within the bacterium's genome. Screening of identified candidates with cell-free transcription-translation systems and in *E. coli* revealed two Acrs, which we named AcrIC11 and AcrIF12*_Xal_*, that inhibit the activity of Cas3 but not Cascade of the respective system. While AcrF12_Xal_ is homologous to AcrIF12, AcrIC11 shares sequence and structural homology with the anti-restriction protein KlcA. These findings help explain tolerance of self-targeting through two CRISPR-Cas systems and expand the known suite of DNA degradation-inhibiting Acrs.

## Graphical Abstract

**Graphical Abstract:**
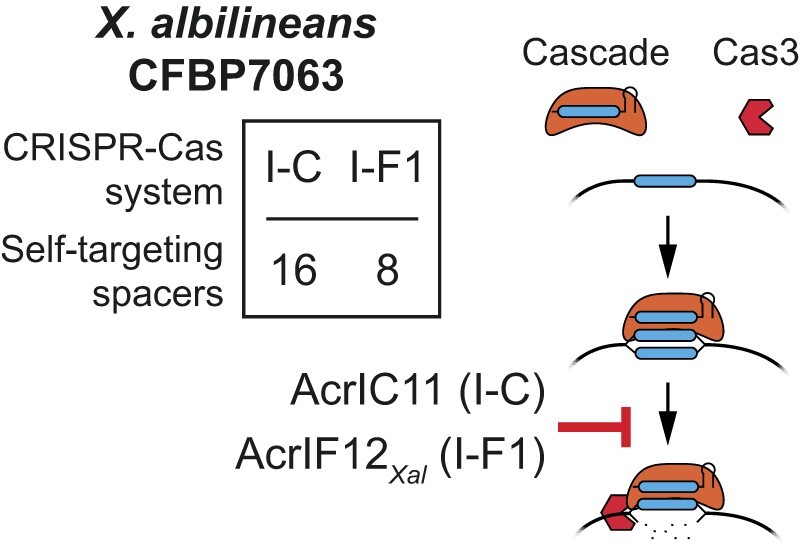

## Introduction

Bacteria and archaea employ a variety of methods to defend against invaders (1). Of these, the only known defenses conferring adaptive immunity are CRISPR-Cas systems. These systems are incredibly diverse, with two classes, six types and >30 subtypes defined to-date (2). Despite their diversity, all CRISPR-Cas systems utilize three general steps for adaptive immunity. In the first step (termed adaptation), CRISPR-Cas systems acquire short nucleic-acid fragments from invaders that are integrated as spacers in between conserved repeats within CRISPR arrays (3,4). In the second step (processing), the CRISPR arrays are transcribed as long precursor CRISPR RNAs (crRNAs) that are processed into mature crRNAs (5). Finally, in the third step (interference), mature crRNAs guide the CRISPR effector proteins to a DNA or RNA region complementary to the spacer portion of the crRNA. Targets flanked by a protospacer-adjacent motif (PAM) or targets lacking complementarity with the repeat portion of the crRNA activate the nuclease (6–9). Activation then leads to either cleavage of the target that clears the invader or widespread collateral RNA cleavage that induces cellular dormancy (10–14).

The incorporation of new spacers during the adaptation step is generally biased towards foreign nucleic acids, although accidental incorporation of genomic fragments can occur (15,16). These genomically-acquired spacers would trigger self-attack against its own genome (i.e. autoimmunity) that should be lethal and therefore selected against (12,15,17); nevertheless, these spacers are quite common, with about 20% of bacteria with a CRISPR-Cas system harboring one or multiple self-targeting spacers (16). To-date, several modes of escape have been identified explaining how bacteria can evade autoimmunity triggered by self-targeting spacers (18). One mode is mutating the *cas* genes to inhibit one or multiple steps of CRISPR-Cas targeting, although this outcome sacrifices the protective function of the CRISPR-Cas system (15,19–21). Another mode is mutating or deleting the target region or flanking PAM to avoid recognition by the CRISPR-Cas system (15,22,23). A third is to block *cas* expression, again sacrificing the protective function. A final mode is inhibiting targeting by the CRISPR-Cas systems through anti-CRISPR proteins (Acrs), small and diverse proteins often encoded in prophages (16), that subvert immune defense but can also prevent autoimmunity.

While the different escape modes avert self-targeting for the affected bacteria, it is hard to determine the exact mechanism in a given bacterium with a known self-targeting system. Sequence-based bioinformatic analyses can identify some escape modes such as mutation of *cas* genes or target regions. However, other modes can be difficult to identify based on sequence information alone. Nevertheless, exploration of bacteria with self-targeting spacers revealed new classes of Acrs and uncovered functions of CRISPR-Cas systems that extend beyond adaptive immunity (24–31). To-date, few experimental investigations of self-targeting have been conducted, with most focusing on bacteria with a single CRISPR-Cas system and few self-targeting spacers (18). In this study, we investigated self-targeting by two type I CRISPR-Cas systems encoded in the plant pathogen *Xanthomonas albilineans* CFBP7063. We discovered two endogenous Acrs that we named AcrIC11 and AcrIF12*_Xal_*, which inhibit the respective system's nuclease activity but not DNA binding activity. Interestingly, AcrIC11 is homologous to the anti-restriction protein KlcA, suggesting that this Acr could also inhibit a distinct and common class of bacterial defenses. Our results uncover how *X. albilineans* likely escapes extensive self-targeting through two orthogonal CRISPR-Cas systems and expand the small set of known Acrs known to inhibit nuclease activity of type I CRISPR-Cas systems.

## Materials and methods

### Plasmid construction

Supplementary Table S1 lists all plasmids used in this work. pXalb_IC_Cascade_GG was produced by Gibson Assembly (GA) using pXalb_IC_Cascade (32) as backbone and adding two type I-C repeats interspaced by *mrfp1* that can be excised with the restriction enzyme SapI. J23108 was used as a promoter driving array expression. pXalb_IC_Cascade_sp1-4 were produced with GoldenGate using pXalb_IC_Cascade_GG as backbone and SapI (NEB) as restriction enzyme. Inserts were ordered from IDT as single-stranded oligos, phosphorylated by T4 PNK (NEB) and annealed by heating to 95°C for 5 min and gradually cooling to room temperature.

pXalb_IF1_Cascade_sp1 was created by GA using pXalb_IF1_Cascade (32) as backbone and adding two type I-F1 repeats interspaced by spacer 1. J23108 was used as a promoter driving array expression. pXalb_IF1_Cascade_sp2-4 were created by Site Directed Mutagenesis (SDM) on pXalb_IF1_Cascade_sp1. pXalb_IC_Cascade_NT and pXalb_IF1_Cascade_NT were created by SDM on pXalb_IC_Cascade_GG and pXalb_IF1_Cascade_sp1, respectively. pXalb_IC_Cas3_J23105 and pXalb_IF1_Cas2-3_J23105 were created by GA using pXalb_IC_Cas3 and pXalb_IF1_Cas2-3 (32) for nuclease amplification and pCB705 (33) as backbone, and changing kanamycin resistance to ampicillin resistance. pXalb_noCas3 was produced with SDM on pXalb_IC_Cas3_J23105. p70a_deGFP_sc101 was created by changing the origin of replication (ori) of p70a_deGFP to sc101 with GA using pCB705 (33) as source for the ori.

pAcr_1–17_T7, pAcrIF12_T7 and pAcrIF3_T7 were created by GA using pET28a as backbone and double stranded DNA fragments containing *E. coli* codon optimized Acr sequences ordered from IDT as inserts. pAcr_1/3/5/7_J23105 and pAcr_15_J23115 were created by SDM on pAcr_1/3/5/7/15_T7, respectively.

All constructed plasmids were verified with Sanger sequencing. Plasmids pXalb_IC_Cas3 and pXalb_IF1_Cas2-3 were previously deposited to Addgene and are available with the plasmid ID 178766 and 178769, respectively.

### RNA Sequencing

*X. albilineans* CFBP7063 (also named GPE PC73) was grown in TSB medium to an OD of 1.0 and 2 mL were pelleted. Total RNA was extracted with Direct-zol RNA Miniprep Plus (Zymo Research) including the in-column DNase I treatment according to manufacturer's instructions. An additional DNase I treatment with TURBO DNA-*free* Kit (Thermo Fisher) was performed and the RNA was cleaned with RNA Clean & Concentrator (Zymo Research). The RNA sample was split into two parts, where one part was used for sequencing of total RNA and the second part was used to sequence shorter-length RNAs.

For sequencing of total RNA, ribosomal RNA was depleted and the cDNA library was prepared using NEBNext Ultra II Directional RNA Library Preparation Kit (NEB). Next-generation sequencing was performed with 50-bp paired-end reads with 25 million reads on an Illumina NovaSeq 6000 sequencer. Sequencing quality was assessed with FastQC (https://www.bioinformatics.babraham.ac.uk/projects/fastqc/) and sequencing data was cleaned with Cutadapt (34). Reads were mapped to the *X. albilineans* CFBP7063 genome (FP565176.1) using RNA STAR (35) and visualized with Geneious Prime 2019.1.3 (https://www.geneious.com). Htseq-count (36) was used to determine the amount of reads per gene for calculation of TPM.

For RNA sequencing of shorter-length RNAs, the cleaned RNA was treated with 2 U/μl T4 PNK (NEB) in 1x T4 DNA Ligase Reaction Buffer (NEB) and 1 U/μl SUPERase•In RNase Inhibitor (Thermo Fisher) for 40 min at 37°C. An additional clean up with RNA Clean & Concentrator (Zymo Research) was added. RNAs with a length of 15–100 nts were selected and the library was prepared using NEBNext Small RNA Library Preparation Kit (NEB). Next-generation sequencing was performed with 150 bp paired-end reads with 30 million reads on an Illumina NovaSeq 6000 sequencer. Sequencing quality was assessed with FastQC (https://www.bioinformatics.babraham.ac.uk/projects/fastqc/) and sequencing data was cleaned with Cutadapt (34). Bowtie2 (37,38) was used to align sequencing data to the *X. albilineans* CFBP7063 genome (FP565176.1) and Geneious Prime 2019.1.3 (https://www.geneious.com) was used to visualize the alignment.

Total RNA-Seq and small RNA-Seq were performed in biological duplicates. The sequencing data is publicly available through GEO accession number GSE229478. Significance between the expression of the target genes and other genes in major prophage regions was calculated using the average TPM values between replicates and using a one-sided Student's t-test with unequal variance. *P* = 0.05 was used as the cutoff for significance.

### Cascade binding assay in *E. coli*

To assess the binding ability of the type I-C CRISPR-Cas system, *E. coli* MG1655 containing p70a_deGFP_sc101 and pXalb_IC_Cascade_s4 or pXalb_IC_Cascade_NT were used. *E. coli* MG1655 with pXalb_IC_Cascade_s4 only were used as negative control. To determine binding ability of type I-F1 CRISPR-Cas system, *E. coli* MG1655 containing p70a_deGFP_sc101 and pXalb_IF1_Cascade_s4 or pXalb_IF1_Cascade_NT were used. *E. coli* MG1655 with pXalb_IF1_Cascade_s4 only were used as negative control.

Cells were grown in appropriate selection medium at 37°C for 16 h. After back diluting cells to OD_600_= 0.02 cells were grown at 37°C to OD_600_= 0.8. Cells were diluted 1:25 in 1xPBS and deGFP fluorescence was measured by flow cytometry using the Accuri C6 Plus analytical flow cytometer (BD Biosciences). Gating on living cells was applied and 30000 events were measured. Final fluorescence values were calculated by subtracting fluorescence obtained from the negative control. Fold-reduction was calculated by the ratio of no-array over the targeting final fluorescence values. Significance was calculated between the no-array and the targeting fluorescence values using Welch's *t*-test. *P* > 0.05 is shown as ns, *P* < 0.05 is shown as *, *P* < 0.01 is shown as ** and *P* < 0.001 is shown as ***.

### Cas3 degradation assay in *E. coli*

To assess degradation ability of the type I-C system, electrocompetent *E. coli* MG1655 containing type I-C Cascade and a targeting array (pXalb_IC_Cascade_sp1-3) or a no-array control (pXalb_IC_Cascade_NT) were prepared and electroporated with 50 ng pXalb_IC_Cas3_J23105. 50 ng pXalb_noCas3 were electroporated as a no-nuclease control. After a one hour recovery in SOC medium at 29°C, samples were diluted 1:100 in LB medium with 34 μg/mL chloramphenicol (Cm) and incubated at 29°C for 16 h. Following, 1:5 dilutions series of the cultures were prepared and 5 μl spot dilutions were plated on LB plates with 34 μg/mL Cm and 100 μg/mL ampicillin (Amp). The plates were incubated at 29°C for 24 h before calculation of colony forming units (CFU) values.

Degradation ability of the type I-F1 system was studied with electrocompetent *E. coli* MG1655 containing the type I-F1 Cascade and a targeting array (pXalb_IF1_Cascade_sp1-3) or a no-array control (pXalb_IF1_Cascade_NT) that were electroporated with 50 ng pXalb_IF1_Cas2-3_J23105. 50 ng pXalb_noCas3 was electroporated as a no-nuclease control. After a one-hour recovery in SOC medium at 37°C, samples were diluted 1:100 in LB medium with 34 μg/mL Cm and incubated at 37°C for 16 h. Following, 1:5 dilutions series of the cultures were prepared and 5 μl spot dilutions were plated on LB plates with 34 μg/mL Cm and 100 μg/mL Amp. The plates were incubated at 37°C for 16 h before calculation of CFU values.

Transformation fold-reduction was calculated by the ratio of no-array CFU values over targeting CFU values. Significance was calculated between the log_10_(CFU) values obtained by the no-array samples and the targeting samples using Welch's *t*-test. *P* > 0.05 is shown as ns, *P* < 0.05 is shown as *, *P* < 0.01 is shown as ** and *P* < 0.001 is shown as ***.

### Acr prediction in *X. albilineans*

To identify Acr candidates in *X. albilineans* CFBP7063, we adopted a two-pronged approach looking for novel Acrs or homologs of known Acrs. In our first approach, we performed a guilt-by-association search for HTH motif-containing proteins that are typically found flanking Acr proteins (39). We then focused on candidates contained in predicted prophage regions on the chromosome based on VirSorter v1.0.3 (53), Prophage Hunter (54) and PHASTER (55,56) as well as on any of the three plasmids. The predicted prophage regions are listed in Supplementary Table S2. In our second approach, we began by assembling a database of Acr protein sequences, host genomes and virus genomes. The final database contains 400 Acr protein sequences, which were derived from downloading Acr proteins from published papers (25,39–47). To define new and extended Acr protein families, we performed an all-against-all sequence similarity comparison on the Acr protein sequences using the Fasta tool (48). Subsequently, we clustered the Acrs using the Markov Cluster Algorithm (MCL) (49) based on previously published similarity criteria (50). Next, we used the MUSCLE tool (51) to generate a multiple sequence alignment (MSA) for each protein cluster. Successively, we created Hidden Markov models (HMMs) for each cluster based on the generated MSA using Hmmbuild (51,52). The HMM profile models were then run against all genes within the *X. albilineans* CFBP7063 genome using the Hmmsearch tool. All hits with an e-value below the cut-off of 0.001 were selected. The final list of putative Acrs can be found in Supplementary Table S3, which includes how the Acrs were predicted.

To find protein sequences homologous to AcrIC11, we performed a comprehensive search using four iterations of PSI-BLAST against the metagenomic and NCBI non-redundant protein databases. Next, multiple sequence alignments of the identified proteins and AcrIC11 were generated with the Muscle tool. Finally, a phylogenetic tree was constructed.

### Acr activity in TXTL Cascade binding assay

The Cas proteins required for Cascade formation that were used in TXTL experiments were encoded on separate plasmids. Therefore, a MasterMix with the required Cas protein encoding plasmids in their stoichiometric amount was prepared beforehand. For the type I-C system, we used a stoichiometry of Cas5_1_-Cas8c_1_-Cas7_7_ and for the type I-F1 system, we used the stoichiometry Cas8f1_1_-Cas5f1_1_-Cas7f1_6_-Cas6f_1_.

To test if and to what extent predicted Acrs lead to inhibition of binding activity in TXTL, we further developed our previously used TXTL deGFP repression assays (32). Therefore, we prepared 3 μl TXTL reactions containing the following: 2.25 μl myTXTL Sigma 70 Master Mix, 0.2 nM p70a_T7RNAP, 0.5 mM IPTG, 1nM pXalb_IC/IF1_gRNA1/nt, 0.5 nM I-C or I-F1 Cascade MasterMix and 1 nM or 0.125 nM pAcr_X_T7 (1 nM: Acr_1–14 and Acr_16; 0.125 nM: Acr_15 and Acr_17). Acr_15 and Acr_17 were added in lower concentrations to avoid unspecific deGFP-inhibition that we observed at a concentration of 1 nM. Reactions without Acr-containing plasmids were used as ‘-Acr’ controls. The TXTL reactions were incubated in a 96-well V-bottom plate at 29°C for 4 h to ensure the formation of a ribonucleoprotein complex. Furthermore, the incubation time leads to expression of the Acrs and allows for inhibition of first steps during CRISPR-Cas activity. After the incubation time, 1 nM p70a_deGFP reporter plasmid is added to the TXTL mix, the reaction is incubated at 29°C for an additional 16 h and fluorescence endpoints are measured with BioTek Synergy H1 plate reader (BioTek) at 485/528 nm excitation/emission (53). The crRNAs encoded in pXalb_IC/IF1_gRNA1 are designed to target within the *degfp* promoter region 3′ of a TTC or a CC PAM for the type I-C or the type I-F1 system, respectively, to ensure active targeting leads to inhibition of deGFP expression. All reactions were prepared with the liquid handling machine Echo525 (Beckman Coulter). Inhibition was calculated with the following equation:


\begin{equation*}{\mathrm{\% }}Inhibition = 100{\mathrm{\% *}}\frac{{\frac{{deGFP\left( {t,Acr} \right)}}{{deGFP\left( {nt,Acr} \right)}} - \frac{{deGFP\left( {t, - Acr} \right)}}{{deGFP\left( {nt, - Acr} \right)}}}}{{1 - \frac{{deGFP\left( {t, - Acr} \right)}}{{deGFP\left( {nt - Acr} \right)}}}}.\end{equation*}

### Acr activity in TXTL Cas3 degradation assay

To test Acrs for their inhibitory activity on type I-C or type I-F1 degradation in TXTL, we extended our previously used degradation assay (32) similar to the above described test to check inhibition of Cascade binding. We shifted the target region from the *degfp* promoter to an upstream sequence (flanked by a 5′ TTC or 5′ CC PAM for the type I-C and the type I-F1 system, respectively). Cas3 was added to the TXTL reaction to enable degradation of the reporter plasmid and thereby reduce deGFP production while Cascade binding without degradation would not impair deGFP expression. Inhibition of a CRISPR-Cas system by an Acr in the degradation test but not in the binding test indicates specific inhibition of DNA degradation by the Acr.

For the initial test analyzing Acr_1–17 3 μl TXTL reactions were prepared. The TXTL reactions including type I-C Cas proteins included the following: 2.25 μl myTXTL Sigma 70 Master Mix, 0.2 nM p70a_T7RNAP, 0.5 mM IPTG, 1 nM pXalb_IC_gRNA2/nt, 0.5 nM pXalb_IC_Cas3, 1 nM I-C Cascade MasterMix and 1 nM or 0.125 nM pAcr_X_T7 (1 nM: Acr_1–14 and Acr_16; 0.125 nM: Acr_15 and Acr_17). Reactions including type I-F1 Cas proteins were composed of: 2.25 μl myTXTL Sigma 70 Master Mix, 0.2 nM p70a_T7RNAP, 0.5 mM IPTG, 1 nM pXalb_IF1_gRNA2/nt, 0.25 nM pXalb_IF1_Cas2-3, 0.5 nM I-F1 Cascade MasterMix and 1 nM or 0.125 nM pAcr_X_T7 (1 nM: Acr_1–14 and Acr_16; 0.125 nM: Acr_15 and Acr_17). TXTL reactions were pre-incubated at 29°C for 4 h. The reporter plasmid p70a_deGFP was added to the reaction to a final concentration of 1 nM and incubated at 29°C for additional 16 h. Fluorescence endpoints are measured with BioTek Synergy H1 plate reader (BioTek) at 485/528 nm excitation/emission (53). All reactions were prepared with the liquid handling machine Echo525 (Beckman Coulter). Inhibition was calculated with the following equation:


\begin{equation*}\% Inhibition = 100\% *\frac{{\frac{{deGFP\left( {t,Acr} \right)}}{{deGFP\left( {nt,Acr} \right)}} - \frac{{deGFP\left( {t, - Acr} \right)}}{{deGFP\left( {nt, - Acr} \right)}}}}{{1 - \frac{{deGFP\left( {t, - Acr} \right)}}{{deGFP\left( {nt, - Acr} \right)}}}}.\end{equation*}

‘nt’ represents values with a non-targeting spacer and ‘t’ represents values with a targeting spacer.

Experiments to assess the inhibitory range of Acr_3 (AcrIC11) were performed as described above with final Acr plasmid concentrations (pAcr_3_T7) ranging from 1 nM to 0.25 nM. Inhibitory range of Acr_1 (AcrIF12*_Xal_*) was investigated with 5 μl TXTL reactions. Thereby, a ‘homemade TXTL’ (54) was used. Type I-F1 Cas proteins, crRNA and Acr_1 were pre-expressed in half the reaction volume. Fresh homemade TXTL including the reporter plasmid was added after the incubation time to prolong activity of the TXTL mix. 2.5 μl pre-expression reactions contained the following: 0.83 μl TXTL extract, 1.04 μl TXTL buffer, 0.4 nM p70a_T7RNAP, 1 mM IPTG, 2 nM pXalb_IF1_gRNA2/nt, 0.5 nM pXalb_IF1_Cas2-3, 2 nM I-F1 Cascade MasterMix and 2–2^−8^ nM pAcr_1_T7. TXTL reactions were pre-incubated at 29°C for 4 h. The following 2.5 μl reactions were added after incubation time: 0.83 μl TXTL extract, 1.04 μl TXTL buffer and 2 nM p70a_deGFP. Both reactions combined resulted in final plasmid concentrations of: 0.2 nM p70a_T7RNAP, 0.5 mM IPTG, 1 nM pXalb_IF1_gRNA2/nt, 0.25 nM pXalb_IF1_Cas2-3, 1 nM I-F1 Cascade MasterMix, 1–2^−9^ nM pAcr_1_T7 and 1 nM p70a_deGFP. The 5 μl reactions were incubated at 29°C for 14 h. All reactions were prepared by hand.

Reactions comparing the inhibitory activity of Acr_1 (AcrIF12*_Xal_*), AcrIF12 and AcrIF3 were performed in 5 μl TXTL reactions as described above. pAcr_1_T7, pAcrIF12_T7 or pAcrIF3_T7 was added at final concentrations of 1–4 nM.

### Acr activity in *E. coli* Cascade binding assay

To test the inhibition of Cascade binding by Acrs in *E. coli*, we adapted our flow cytometry assay assessing binding ability. *E. coli* MG1655 containing the reporter plasmid p70a_deGFP_sc101, pAcr_1/3/5/7_J23105, pAcr_15_J23115 or pET28a (‘-Acr’ control) and pXalb_IC_Cascade_s4 or pXalb_IC_Cascade_NT were used to investigate the type I-C system. *E. coli* MG1655 with pXalb_IC_Cascade_s4 only were used as negative control. To determine binding ability of the type I-F1 CRISPR-Cas system, *E. coli* MG1655 containing p70a_deGFP_sc101, pAcr_1/3/5/7_J23105 or pAcr_15_J23115 and pXalb_IF1_Cascade_s4 or pXalb_IF1_Cascade_NT were used. *E. coli* MG1655 with pXalb_IF1_Cascade_s4 only were used as negative control.

Cells were grown in appropriate selection medium at 37°C for 16 h. After back diluting cells to OD_600_= 0.02 cells were grown at 37°C to OD_600_= 0.8. After cells were diluted 1:25 in 1xPBS, deGFP fluorescence was measured by flow cytometry using the Accuri C6 Plus analytical flow cytometer (BD Biosciences). Gating on living cells was applied and 30 000 events were measured. Final fluorescence values were calculated by subtracting fluorescence obtained from the negative control. deGFP fold-repression was calculated by the ratio of no-array over the targeting final fluorescence values. Significance was calculated between the -Acr samples and the Acr-containing samples using Welch's *t*-test. *P* > 0.05 is shown as ns, *P* < 0.05 is shown as *, *P* < 0.01 is shown as ** and *P* < 0.001 is shown as ***. Inhibition was calculated with the following equation:


\begin{equation*}\% Inhibition = 100\% *\frac{{\frac{{deGFP\left( {T,Acr} \right)}}{{deGFP\left( {NT,Acr} \right)}} - \frac{{deGFP\left( {T, - Acr} \right)}}{{deGFP\left( {NT, - Acr} \right)}}}}{{1 - \frac{{deGFP\left( {T, - Acr} \right)}}{{deGFP\left( {NT, - Acr} \right)}}}}\end{equation*}

‘NT’ represents no-array values and ‘T’ represents targeting final values.

### Acr activity in *E. coli* Cas3 degradation assay

To test the activity of Acrs in degradation inhibition in *E. coli*, we adapted our transformation assay assessing degradation ability. For the type I-C system, electrocompetent *E. coli* MG1655 containing type I-C Cascade, a targeting array (pXalb_IC_Cascade_sp2) or a no-array control (pXalb_IC_Cascade_NT), and pAcr_1/3/5/7_J23105, pAcr_15_J23115 or pET28a (‘-Acr’ control) were prepared and electroporated with 50 ng pXalb_IC_Cas3_J23105. After a one hour recovery in SOC medium at 29°C, samples were diluted 1:100 in LB medium with 34 μg/mL Cm and 50 μg/mL kanamycin (Kan) and incubated at 29°C for 16 h. Following, 1:5 dilutions series of the cultures were prepared and 5 μl spot dilutions were plated on LB plates with 34 μg/mL Cm, 50 μg/ml Kan and 100 μg/ml Amp. The plates were incubated at 29°C for 24 h before calculation of CFU values.

Degradation ability of the type I-F1 system was studied with electrocompetent *E. coli* MG1655 containing the type I-F1 Cascade, a targeting array (pXalb_IF1_Cascade_sp3) or a no-array control (pXalb_IF1_Cascade_NT), and pAcr_1/3/5/7_J23105, pAcr_15_J23115 or pET28a (‘-Acr’ control) that are electroporated with 50 ng pXalb_IF1_Cas2-3_J23105. After a one hour recovery in SOC medium at 37°C, samples were diluted 1:100 in LB medium with 34 μg/ml Cm and 50 μg/ml Kan and incubated at 37°C for 16 h. Following, 1:5 dilutions series of the cultures were prepared and 5 μl spot dilutions were plated on LB plates with 34 μg/ml Cm, 50 μg/ml Kan and 100 μg/ml Amp. The plates were incubated at 37°C for 16 h before calculation of CFU values.

Transformation fold-reduction was calculated by the ratio of no-array over the targeting CFU values. Significance was calculated between the values obtained by the -Acr samples and the Acr-containing samples using Welch's *t*-test. *P* > 0.05 is shown as ns, *P* < 0.05 is shown as *, *P* < 0.01 is shown as ** and *P* < 0.001 is shown as ***. Inhibition was calculated with the following equation:


\begin{equation*}{\mathrm{\% }}Inhibition = 100{\mathrm{\% *}}\frac{{\frac{{CFU\left( {T,Acr} \right)}}{{CFU\left( {NT,Acr} \right)}} - \frac{{CFU\left( {T, - Acr} \right)}}{{CFU\left( {NT, - Acr} \right)}}}}{{1 - \frac{{CFU\left( {T, - Acr} \right)}}{{CFU\left( {NT, - Acr} \right)}}}}\end{equation*}

‘NT’ represents no-array values and ‘T’ represents targeting final values.

### Amino-acid sequence alignment

Amino-acid sequences were aligned with Clustal-Omega 1.2.4. (55).

### AlphaFold prediction and structural comparison

Protein structures of AcrIC11 in Supplementary Figure S3 were predicted using AlphaFold2 available through ColabFold (56,57) with default settings and subsequently visualized by Molstar (58). The structure of KlcA is available through the PDB database (2KMG) (www.RCSB.org) (42). The overlay of the predicted protein structure of AcrIC11 with the highest rank and KlcA was performed using the PyMOL Molecular Graphics System, Version 2.5.5 Schrödinger, LLC. For this, both protein structures were opened in PyMOL, and then KlcA was aligned to AcrIC11, which also calculates the RMSD value. Structural similarity was calculated using TM-align (version 20190922) (59). For comparison of amino acid sequences of AcrIC11 and KlcA, ClustalW multiple sequence alignment by MUSCLE (version 3.8) was performed (51,60).

## Results

### The two self-targeting CRISPR-Cas systems in *Xanthomonas albilineans* are actively expressed

**Figure 1.**
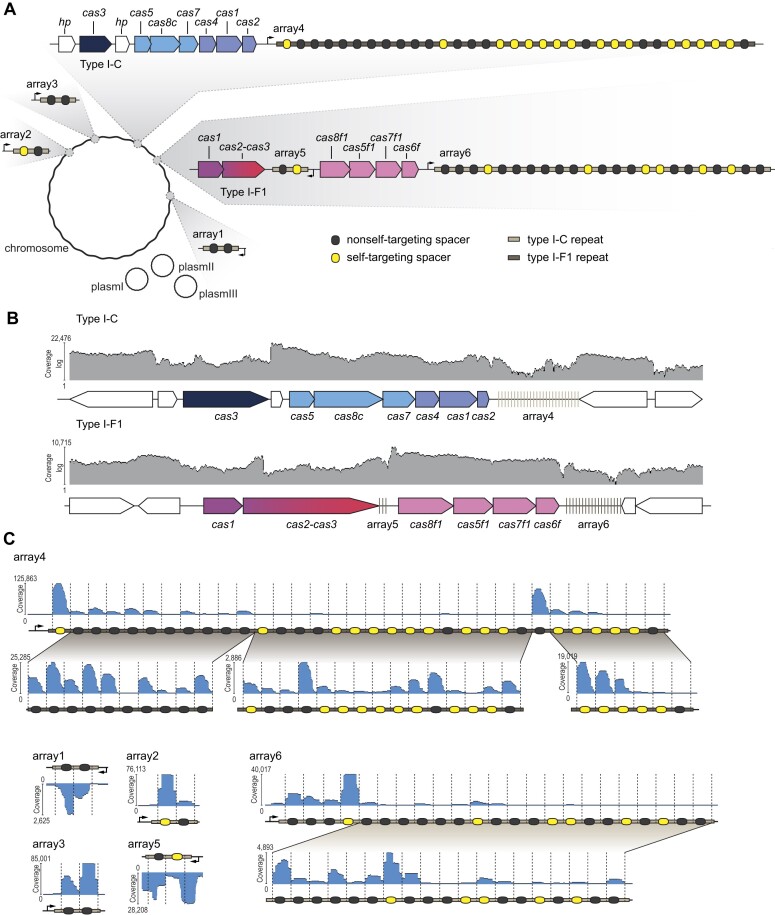
RNA-Seq analysis reveals transcription of *cas* genes and crRNA biogenesis for the two CRISPR-Cas systems in *Xanthomonas albilineans*. (**A**) Overview of the type I-C and type I-F1 CRISPR-Cas systems endogenous to *X. albilineans*. *cas* genes associated with the I-C system and the I-F1 system are shown in different shades of blue and pink, respectively. Spacers complementary to a region in the chromosome of *X. albilineans* or one of its plasmids are shown in yellow, and spacers without complementarity are depicted in black. (**B**) Mapped reads of the type I-C and I-F1 *cas* genes following total RNA-Seq. (**C**) Mapped reads of the mature crRNAs following small RNA-Seq of shorter-length RNAs. As the coverage is highly varying, below each coverage plot we show separate coverage plots for the regions with lower coverage. Expected processing patterns are indicated with dashed lines.

The *Xanthomonas albilineans* strain CFBP7063 encodes two CRISPR-Cas systems, a type I-C system and a type I-F1 system, along with six CRISPR arrays. Of these arrays, one is associated with the type I-C system and five are associated with the type I-F1 system (Figure 1A). In total, four of the six CRISPR arrays (one type I-C array and three type I-F1 arrays) encode 24 self-targeting spacers directed mainly towards predicted prophage regions in the chromosome or towards one of the three plasmids of *X. albilineans* (Supplementary Table S4). Spacers guide their associated effector complex to complementary targets, resulting in target degradation during the interference step of the CRISPR-Cas immunity. As part of interference, the effector complex Cascade (CRISPR-associated complex for antiviral defense), consisting of three to five Cas proteins and the mature crRNA, binds to the target DNA (61). The endonuclease Cas3 is then recruited to the target bound by Cascade (62) to nick the non-target strand and degrade the DNA in a 3′-to-5′ direction (63–65). Our previous work showed that both systems from *X. albilineans* efficiently carried out both steps of type I interference (32).

If *cas* genes are functionally encoded, one mode to evade lethal self-targeting is preventing expression of all or some *cas* genes. Therefore, we performed RNA sequencing (RNA-Seq) analysis on *X. albilineans* under different growth conditions. We could detect transcripts for all 13 *cas* genes (Figures 1B and Supplementary S1), with expression levels ranging between 7 TPM (transcripts per million) for type I-F1 *cas2-3* and 910 TPM for type I-C *cas5*. To compare these values to genes that should be functionally expressed, we depicted ten genes that were found to be essential in a member of the *Xanthomonas* species and exhibit TPM levels comparable to the I-C and I-F1 *cas* genes (66) (Supplementary Figure S1). Thus, *X. albilineans* does not appear to protect against lethal self-targeting by actively suppressing transcription of the *cas* genes.

Expected processing of pre-crRNAs to mature crRNAs would be another indication of functional expression of the Cas proteins, as Cascade proteins are required for processing of crRNAs and the stability of mature crRNAs (67,68). To examine crRNA processing, we performed RNA-Seq analysis on shorter-length RNAs (Figure 1C). Most spacers in the CRISPR arrays gave rise to the expected mature crRNAs for either system, while the short arrays 1 and 5 yielded atypical processing products (Figures 1C and Supplementary S1) (5). Array 4, the sole type I-C associated array, generally yielded in the expected 11-nt 5′ handle (Figures 1C and Supplementary S1), while arrays 2, 3 and 6 yielded the expected processing pattern of type I-F1 systems (8-nt 5′ handle) (Figures 1C and Supplementary S1) (5). Interestingly, in arrays 2, 4 and 6, the most abundant crRNAs were self-targeting crRNAs (Figure 1C), excluding the possibility of preventing autoimmunity by solely expression of crRNAs targeting foreign DNA. Therefore, we conclude that mature crRNAs as well as the necessary Cas proteins are produced to elicit self-targeting in *X. albilineans*.

### Both CRISPR-Cas systems bind and degrade target DNA in *E. coli*

Beyond *cas* expression and crRNA processing, we investigated interference as the last step of CRISPR-Cas immunity. While interference could not be assessed in *X. albilineans* due to technical issues with plasmid transformation, our prior testing of Cascade and Cas3 with cell-free transcription-translation (TXTL) systems suggested that the I-C and I-F1 CRISPR-Cas systems each could enact interference in isolation (32). To assess if interference activity could lead to lethal chromosomal degradation, we assessed DNA targeting by either system in *E. coli*.

**Figure 2.**
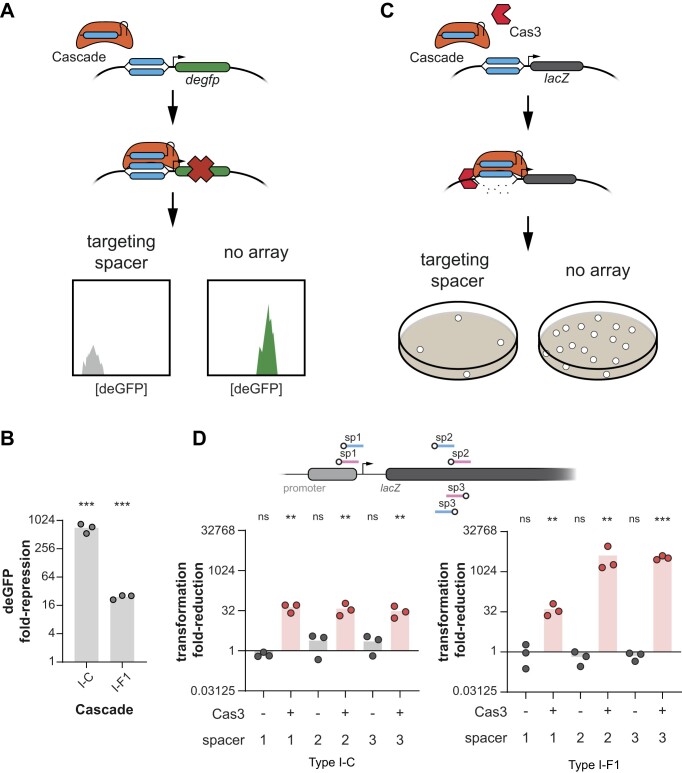
The type I-C and I-F1 CRISPR-Cas systems from *X. albilineans* bind and degrade target DNA in *E. coli*. (**A**) Overview of the DNA binding assay in *E. coli*. Cascade (orange) is guided by its crRNA to the target region (blue) on the deGFP-reporter plasmid complementary to the spacer (blue). Cascade binding to its target covering the promoter of *degfp* inhibits deGFP expression that can be measured by flow cytometry. The experimental setup lacking a CRISPR array (no array) serves as a negative control. (**B**) DNA binding by Cascade from both *X. albilineans* systems in *E. coli*. Without Cas3, Cascade can only bind target DNA, repressing deGFP expression but without target cleavage. (**C**) Overview of the DNA degradation assay in *E. coli*. Cascade (orange) is guided by its crRNA to the target region (blue) within the promoter or the coding region of *lacZ* on the *E. coli* chromosome (target locations are shown in D) and recruits Cas3 (red). CRISPR-Cas interference causes DNA degradation which reduces the colony count on agar plates (gray). The experimental setups lacking a CRISPR array (no array) or lacking Cas3 serve as negative controls. (**D**) DNA degradation by Cascade and Cas3 from both *X. albilineans* systems in *E. coli*. Fold-reduction in B and D is calculated based on a no-array control that is missing a spacer complementary to the *E. coli* genome or the reporter plasmid. The no-array control is the reference for statistical analyses. Bars indicate the mean of triplicate independent experiments. ***P < 0.001. **P < 0.01. *P < 0.05. ns: *P* > 0.05.

As Cascade must bind its DNA target before recruiting the nuclease Cas3 to induce target degradation (62), we first investigated target binding by Cascade in the absence of Cas3 based on transcriptional repression (69,70). We encoded the associated genes forming Cascade for the I-C system (*cas5*, *cas8c* and *cas7*) or the I-F1 system (*cas8f1*, *cas5f1*, *cas7f1* and *cas6f*) as an operon on a plasmid under a constitutive promoter. The same plasmid also encoded a constitutively expressed single-spacer array we used in a previous study to target the deGFP reporter plasmid (32). The targets in the promoter of *degfp* were flanked by a 5′ TTC (I-C system) or 5′ CC (I-F1 system) PAM, which we previously identified and validated as preferred PAMs *in vitro* (32). Finally, the targeted deGFP reporter plasmid was added, and deGFP production was measured (Figure 2A). Cascade of both CRISPR-Cas systems repressed deGFP expression by ∼700-fold (I-C system) and ∼25-fold (I-F1 system) compared to the non-targeting control (Figure 2B). Therefore, either system's Cascade can bind DNA targets *in vivo*.

As target binding was successfully performed by the I-C and the I-F1 Cascade, we proceeded to test targeted DNA degradation by Cas3. We exchanged the deGFP reporter plasmid with a plasmid encoding the I-C *cas3* or the I-F1 *cas2-3*. We then tested three different spacers targeting the promoter or the coding region of the chromosomal gene *lacZ* with a flanking 5′ TTC (I-C system) or 5′ CC (I-F1 system) PAM (Figures 2C and D). Both CRISPR-Cas systems significantly reduced plasmid transformation compared to the no-array control, indicating chromosomal degradation and cell death (Figures 2C and D). All three spacers of the type I-C system similarly reduced plasmid transformation, whereas spacer 2 and spacer 3 exhibited an ∼80–100 times higher fold change than spacer 1 in the type I-F1 system (Figure 2D). As expected, the absence of Cas3 negligibly reduced plasmid transformation in both systems (Figure 2D). Given the lethality of chromosomal targeting with Cascade and Cas3 from either system, additional factors separate from Cascade and Cas3 likely exist that protect the *X. albilineans* from lethal self-targeting.

### Predicted anti-CRISPR proteins inhibit both CRISPR-Cas systems in TXTL

**Figure 3.**
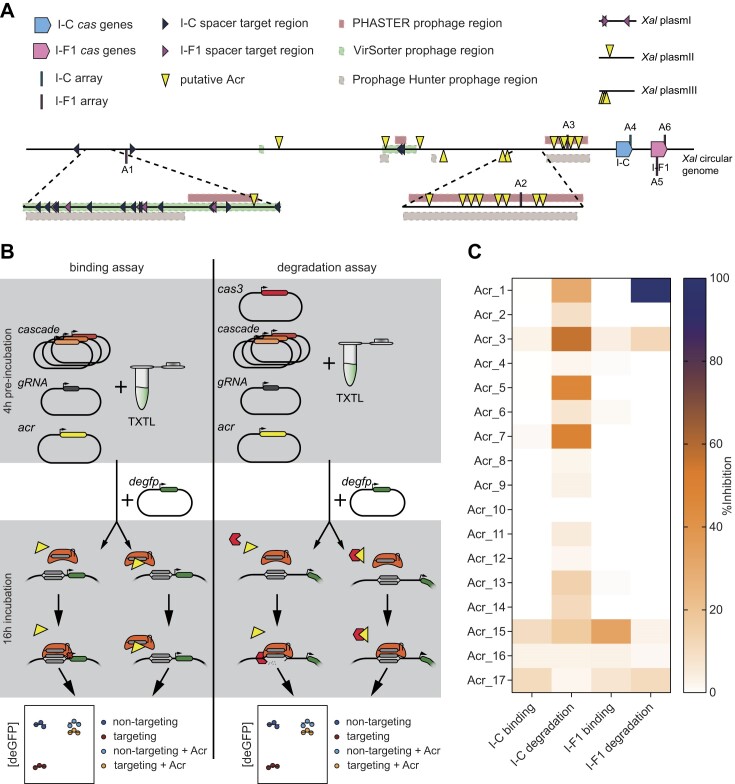
Putative Acrs inhibit DNA binding or DNA degradation via either *X. albilineans* CRISPR-Cas system in TXTL. (**A**) Overview of the genomic organization of CRISPR-Cas systems, putative Acrs and predicted prophages regions in *X. albilineans*. The numbering of the arrays corresponds to that in Figure 1. Placement of the arrays, Acr candidates and self-targets indicates whether they are encoded on the top or bottom strand of the chromosome or plasmid. Prophage regions are predicted with VirSorter v1.0.3 (73), Prophage Hunter (74) and PHASTER (75,76). Amino-acid sequences of all Acr candidates and their genomic location in *X. albilineans* can be found in Supplementary Table S3. (**B**) Overview of testing Acr candidates for their binding and degradation inhibition in TXTL. On the left side, inhibition of binding activity is tested. Inhibition of Cascade-mediated transcriptional repression of deGFP expression indicates a functional Acr. On the right side, inhibition of degradation activity is assessed. Inhibition of DNA degradation by Cas3 recruited by Cascade indicates a functional Acr. Inhibition of DNA degradation while allowing Cascade-mediated DNA-binding classifies an Acr as a degradation-inhibiting Acr. (**C**) Inhibitory activity of putative Acrs in TXTL. Inhibitory activity of Acr candidates was tested in triplicates and the mean inhibitory activity is depicted.

We hypothesized that lethal self-targeting by both CRISPR-Cas systems is inhibited by the presence of Acrs encoded within the *X. albilineans* CFBP7063 genome (71,72). To identify potential Acrs, we performed a two-pronged approach by searching for homologs of known Acrs as well as applying guilt-by-association (39) that evaluates genes flanking HTH-containing genes and that are found within predicted prophage regions (73–76) or on any of the three plasmids (see Figure 3A, Supplementary Table S2 and methods). This search produced 17 Acr candidates (initially named Acr_1 through Acr_17) (Supplementary Table S3). Our RNA-Seq analyses indicated that a subset of the predicted Acrs is expressed in *X. albilineans* (Supplementary Table S2), suggesting at least some of the Acr candidates might actively inhibit one or both CRISPR-Cas systems.

We first subjected the predicted Acrs to TXTL assays we used previously (33,77) to assess their inhibitory activity. TXTL assays involve adding DNA constructs, resulting in the production of the encoded RNAs and proteins whose activity can be evaluated in the same reaction. We specifically developed two assays to evaluate the extent to which the inhibitory activity of each predicted Acr acted on or upstream of DNA binding, or on or upstream of DNA degradation (Figure 3B). The first assay is an extension of the previously used DNA binding assay and assesses inhibition of Cascade-mediated transcriptional repression of deGFP expression. Active Acrs prevent binding of Cascade to a target in the *degfp* promoter, resulting in unhindered deGFP expression. The second assay is an extension of the previous degradation assay and assesses inhibition of DNA degradation by Cas3 recruited by Cascade. Here, a target upstream of the *degfp* promoter is chosen such that active Acrs prevent plasmid degradation. Inhibitory activity in both assays would indicate an inhibitory mechanism at or upstream of DNA binding, while inhibitory activity in only the second assay would indicate a degradation-inhibiting mechanism.

We tested all 17 putative Acrs with both assays for their activity against the type I-C and the I-F1 CRISPR-Cas systems (Figure 3C). Transcriptional repression of *degfp* by the I-C Cascade was not substantially inhibited by any tested Acr candidate, at least not with an inhibitory activity higher than 11%. Type I-C degradation on the other hand was repressed by multiple Acr candidates, with Acr_3 exhibiting the highest inhibitory activity (∼57%) followed by three other Acrs (Acr_1, Acr_5, Acr_7) exhibiting lower but measurable inhibitory activity. Acr_1 fully inhibited degradation by the I-F1 Cas3 but not binding by the I-F1 Cascade, suggesting that this candidate functions as a DNA degradation-inhibiting Acr. Acr_15 partially inhibited repression of deGFP expression in the type I-F1 binding assay by ∼30%, although no inhibition was observed in the degradation assay. No appreciable inhibition was observed for the other Acr candidates suggesting that they are not inhibitors of either system, although we cannot rule out the possibility that the corresponding proteins were not functionally expressed in TXTL.

### Acr_3 and Acr_1 inhibit DNA degradation by the I-C and I-F1 Cas3, respectively, in *E. coli*

**Figure 4.**
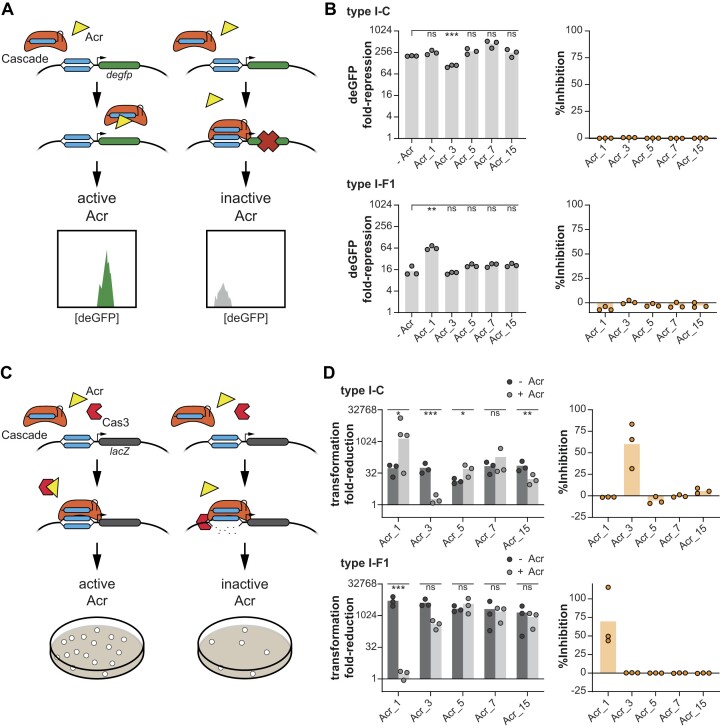
Acr_1 and Acr_3 inhibit DNA degradation but not DNA binding via either *X. albilineans* CRISPR-Cas system in *E. coli*. (**A**) Overview of testing Acrs for inhibition of transcriptional repression by Cascade in *E. coli*. deGFP expression is restored when an Acr actively inhibits at and upstream of Cascade binding to its DNA target. See Figure 2A for more details. (**B**) Inhibitory activity of putative Acrs on Cascade-binding in *E. coli*. deGFP repression was measured with flow cytometry. Bars represent the average of three biological replicas. (**C**) Overview of testing Acrs for inhibition of DNA degradation in *E. coli*. Acrs actively inhibiting any step upstream of and including Cas3-mediated DNA degradation restore transformation efficiency. See Figure 2C for more details. Type I-C spacer 2 and type I-F1 spacer 3 were used here. (**D**) Inhibitory activity of putative Acrs on Cas3-mediated DNA degradation in *E. coli*. Fold-reduction in B and D is calculated based on a no-array control that is missing a spacer complementary to the *E. coli* genome or the reporter plasmid. The -Acr control is the reference for statistical analyses. Bars in B indicate the mean of biological triplicates, while bars in D indicate the mean of biological triplicates carried out with technical triplicates. Data points in B represent biological independent experiments and data points in D represent the mean of technical triplicates of a biologically independent sample. ***P < 0.001. **P < 0.01. *P < 0.05. ns: *P* > 0.05.

Given the fact that Acr_1, Acr_3, Acr_5, Acr_7 and Acr_15 exhibited notable inhibitory activity in TXTL, we next tested these putative Acrs in *E. coli*. Inhibition of DNA binding by Cascade was investigated by adding each Acr candidate to the DNA binding assay and measuring each Acr's ability to inhibit transcriptional repression of deGFP (Figure 4A). Acr_3 significantly but modestly reduced deGFP fold-repression by the I-C Cascade (Figure 4B). All other tested Acr candidates did not significantly reduce deGFP fold-repression for the I-C or the I-F1 Cascade. The lack of binding inhibition in *E. coli* was expected for Acr_1, Acr_3, Acr_5 and Acr_7 given our prior TXTL results (Figure 3C).

As Cascade bound to target DNA recruits Cas3 to induce DNA degradation, we measured the inhibitory activity of each Acr candidate in the *E. coli* DNA degradation assay (Figure 4C). In this setup, inhibition of Cas3-mediated chromosomal DNA degradation would result in elevated colony numbers. Similar to our previous *in vitro* experiments, inhibition of a CRISPR-Cas system in the DNA degradation assay but lacking restoration of deGFP expression in the binding assay categorized the Acr as a degradation-inhibiting Acr. Acr_3 and Acr_1 significantly reduced transformation fold-reduction of the type I-C and type I-F1 system, respectively, compared to a no-Acr control (Figure 4D). Mirroring our TXTL results, Acr_3 inhibited DNA degradation by 60%, while Acr_1 inhibited DNA degradation by 70% (Figure 4D). Furthermore, Acr_15 modestly but significantly reduced plasmid transformation of the I-C Cas3 (17-fold reduction of plasmid transformation compared to 71-fold reduction in the no-Acr control), leaving open the question whether Acr_15 represents a bona fide Acr. All other tested Acr candidates did not substantially suppress degradation of one or both CRISPR-Cas systems. Acr_1 and Acr_3, the two candidates that emerged as validated CRISPR inhibitors, were respectively expressed the highest and third highest amongst the 17 candidates in *X. albilineans* (Supplementary Table S3), in line with active inhibition of self-targeting by both CRISPR-Cas systems. The lack of robust inhibition by Acr_5, Acr_7 and Acr_15 in *E. coli* reflects some disconnect between the experimental setups and underscores how TXTL results require follow-on validation in cellular systems.

**Figure 5.**
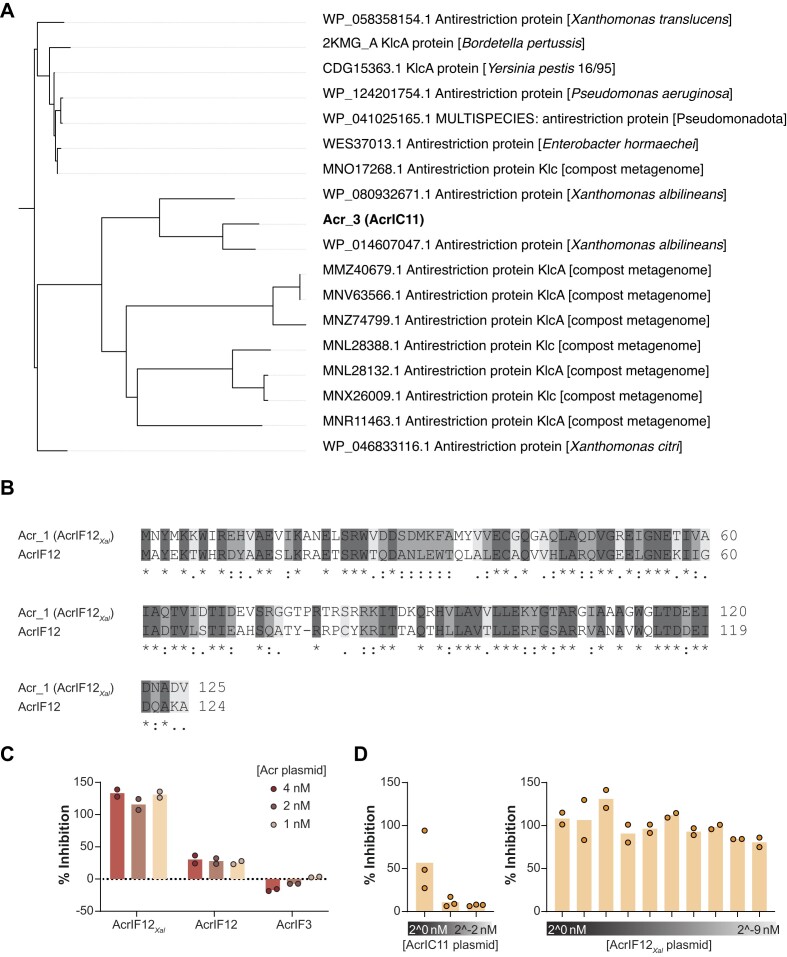
Acr_1 (AcrIF12*_Xal_*) and Acr_3 (AcrIC11) are homologous to AcrIF12 and the anti-restriction protein KlcA. (**A**) Phylogenetic distribution of identified homologs of the Acr candidate Acr_3. Tree branches show close and distant homologs of the Acr candidate. (**B**) Sequence alignment of Acr_1 and AcrIF12. Amino acid sequences of Acr_1 and AcrIF12 were aligned with Clustal-Omega 1.2.4. (55). (**C**) Inhibitory activity of AcrIF12*_Xal_*, AcrIF12 and AcrIF3 on Cas3-mediated DNA degradation in TXTL. Degradation assays were conducted with Acr plasmid concentrations ranging from 4 nM to 1 nM. For more details see Figure 3B. (**D**) Inhibitory activity of AcrIC11 and AcrIF12_Xal_ on Cas3-mediated DNA degradation in TXTL with different Acr concentrations. Degradation assays were conducted with Acr plasmid concentrations ranging from 2^0^ to 2^−2^ nM or 2^0^ to 2^−9^ nM for AcrIC11 or AcrIF12_Xal,_ respectively. Bars in C and D indicate the mean inhibitory activity of triplicate or duplicate independent experiments.

With the validation of the inhibitory activity of Acr_3 and Acr_1 in TXTL and *E. coli*, we asked how both Acrs are related to formerly identified Acrs. Acr_3 does not share high amino-acid similarity to any previously characterized Acr but has numerous closely and distantly related homologs found in Xanthomonads and other bacterial pathogens (Figures 5A and S2). Thus, we renamed Acr_3, following the common nomenclature, to AcrIC11 (78). Interestingly, the identified homologs are either anti-restriction proteins or specifically KlcA, a known inhibitor of Type I restriction-modification systems (79). To further explore the relationship between AcrIC11 and KlcA, we predicted the structure of AcrIC11 using AlphaFold (56,57) and compared it to the published crystal structure of KlcA (79). Based on this comparison, the two proteins adopt similar structures (RMSD = 1.467 Å, TM-score = 0.79111) (59). These findings offer the intriguing possibility that AcrIC11 evolved from an inhibitor of a distinct defense system and may even inhibit both defenses.

Beyond Acr_3, Acr_1 shares 44.8% amino-acid identity with the previously published AcrIF12 (25) (Figure 5B), therefore, we renamed Acr_1 to AcrIF12*_Xal_*. AcrIF12 was discovered next to an anti-CRISPR-associated gene 4 (*aca4*) by the ‘guilt-by-association’ method in *Pseudomonas aeruginosa* (25). The exact mechanism of AcrIF12 is unknown, although it was reported that this Acr does not strongly bind to Cascade nor Cas3 in isolation (80). To test, if AcrIF12 is also active against the *X. albilineans* type I-F1 system, we subjected AcrIF12 to our degradation-assay in TXTL (Figures 3B and 5C). AcrIF12 yielded an inhibitory activity of ∼30%. Interestingly, the inhibitory activity of AcrIF12 and AcrIF12*_Xal_* was maintained when decreasing the Acr plasmid concentration by a factor of four (Figure 5C), where decreasing the plasmid concentration of AcrIF12*_Xal_* by 500-fold dropped the inhibitory activity from ∼100% to only 80% (Figure 5D). In contrast, the inhibitory activity of AcrIC11 dropped from 57% to 11% even when using half the amount of Acr plasmid (Figure 5D). Such inhibitory activities over a wide range of Acr concentrations have been associated with catalytic Acrs (80–82). We also tested a separate Acr shown to specifically inhibit Cas3 from the I-F1 system in *Pseudomonas aeruginosa* as a control (83,84), although no inhibition was observed possibly due to limited host range (Figure 5C). Overall, these results show that *X. albilineans* encodes two Acrs that can actively inhibit DNA degradation but not DNA binding by either CRISPR-Cas system, likely explaining how this bacterium evades self-targeting by two distinct self-targeting systems.

## Discussion

In this study, we identified two degradation-inhibiting Acrs endogenous to *X. albilineans*, which we named AcrIC11 and AcrIF12*_Xal_*. By blocking DNA degradation by Cas3, both Acrs are expected to prevent lethal self-targeting by the two CRISPR-Cas systems in *X. albilineans*. The possibility also remains that additional Cascade-inhibiting Acrs are encoded in the genome of *X. albilineans*. AcrIC11 and AcrIF12_Xal_ add to a growing number of Acrs that inhibit Cas3 but not Cascade by two general mechanisms (25,40,44,47,83–89). AcrIF3 and AcrIE1 directly bind Cas3, while AcrIC3 is suggested to do the same (47,83–85,87). In contrast, AcrIE2 and AcrIF5 bind Cascade and likely block Cas3 recruitment while preserving Cascade-induced DNA-binding (88,89). The mechanisms employed by AcrIC1, AcrIF16 and AcrIF17 to block DNA degradation remain unknown.

A search for AcrIC11 homologs revealed a large set of proteins annotated as the anti-restriction protein KlcA. KlcA was previously identified as an inhibitor of the four main families of Type I restriction-modification systems *in vivo* (79). However, KlcA was unable to inhibit restriction by an archetypal Type I restriction endonuclease *in vitro* and did not resemble standard anti-restriction proteins functioning as DNA mimics (79), indicating that KlcA operates through a distinct mode of action. We further showed that AcrIC11 is predicted to fold into a structure strongly resembling that of KlcA, suggesting overlapping functions (Supplementary Figure S3). What remains to be explored is whether AcrIC11 can inhibit Type I restriction-modification systems and/or KlcA proteins can inhibit Type I-C CRISPR-Cas systems. If so, AcrIC11 would be added to a small but growing list of anti-defense proteins that inhibit multiple bacterial defenses. These include the T7 phage protein Ocr that acts through DNA mimicry to inhibit restriction-modification systems and BREX systems (90,91), as well as phage-encoded nucleotidases that sequester or degrade cyclic nucleotide signaling molecules to inhibit diverse bacterial defenses (92–95). In these examples, though, the anti-defense protein acts through an obvious shared mechanism. In contrast, the most obvious shared property for DNA degradation by the I-C CRISPR-Cas system and DNA restriction by Type I restriction-modification systems is DNA binding, although this is the unlikely mode of inhibition based on AcrIC11 inhibiting DNA degradation but not binding and KlcA failing to block DNA restriction *in vitro*. Thus, further exploring the mechanism of inhibition of AcrIC11 and the extent to which it can also inhibit restriction-modification systems could reveal new means by which individual proteins could circumvent multiple defenses posted by a bacterial host.

Elucidating the exact mechanisms by which AcrIC11 and AcrIF12*_Xal_* inhibit DNA degradation could reveal new mechanisms of action. In particular, the inhibitory mechanism of AcrIF12*_Xal_* and its homolog AcrIF12 likely differs from already known type I degradation-inhibiting mechanisms based on two observations. First, AcrIF12 did not co-elute with Cascade nor Cas3 *in vitro* in a previous study (80), ruling out direct binding with either. Second, we showed that AcrIF12*_Xal_* maintained its inhibitory activity even when its expression plasmid was diluted by 500-fold (Figure 5D), suggesting that AcrIF12*_Xal_* and AcrIF12 could function as multi-turnover proteins. Elucidating the inhibitory mechanism of AcrIF12*_Xal_* and AcrIF12 therefore could reveal unique means by which Acrs inhibit Cas3-mediated DNA degradation.

Inhibition of DNA degradation by AcrIC11 and AcrIF12*_Xal_* still allows for DNA binding and bears the potential to transform each respective CRISPR-Cas system into a gene regulator. By silencing deGFP expression, we demonstrated that Cascade-mediated gene repression is possible even when AcrIC11 and AcrIF12*_Xal_* are present (Figure 2B). Gene regulation by self-targeting spacers can be beneficial as was shown previously in *Francisella novicida* which utilize scaRNAs (small CRISPR/Cas-associated RNAs) to facilitate immune escape during host invasion (29) as well as in *Haloarcula hispanica* which utilize a separately-encoded crRNA to repress expression of a toxin (26). Interestingly, of the six most highly expressed self-targeting crRNAs (array 2: spacer 1; array 4: spacer 1, spacers 28–30; array 6: spacer 4), five are complementary to regions within the first predicted prophage (Figures 1C and 3A, Supplementary Table S4). Furthermore, only one of the spacers, Array 4: spacer 29, would not be expected to yield target DNA binding, as the target region possesses 9 mismatches and a PAM (GGG) not recognized by the I-C system (32). Exploring the potential of gene repression using the RNA-seq dataset, target genes within the main prophage regions were expressed significantly lower than the other genes in these regions (respective average TPM = 23 and 268, *P* = 0.000197) (Supplementary Tables S4–S6). However, more exploration is needed to elucidate the extent to which the self-targets are silenced by either CRISPR-Cas system.

The genomic location of AcrIC11 and AcrIF12_Xal_ provides hints about the history of *X. albilineans*. AcrIF12*_Xal_* is encoded in the first predicted prophage that also harbors many self-targets (16 in total); this would suggest that AcrIF12*_Xal_* facilitated prophage integration by hindering DNA degradation by the type I-F1 system. In contrast, AcrIC11 is encoded on plasmid II that does not harbor any self-targets. We suspect that plasmid II was present in *X. albilineans* before integration of the AcrIF12*_Xal_*-bearing prophage, as the prophage contains multiple targets of the I-C system that would be blocked by the action of AcrIC11. Self-targeting spacers could also be acquired after prophage integration, although this seems unlikely given that many of the self-targeting spacers are located at the older end of their respective CRISPR arrays (96). Overall, elucidating the order of events could shed light on how prokaryotes come to possess self-targeting spacers and the impact on the evolutionary trajectory of each microorganism.

## Supplementary Material

gkad1097_Supplemental_Files

## Data Availability

The NGS data underlying this article is available in the Gene Expression Omnibus (GEO) at https://www.ncbi.nlm.nih.gov/geo/ under accession number GSE229478. Further source data and constructs will be shared on reasonable request to the corresponding author.

## References

[1] Bernheim A. , Sorek R. The pan-immune system of bacteria: antiviral defence as a community resource. Nat. Rev. Microbiol. 2020; 18:113–119. 31695182 10.1038/s41579-019-0278-2

[2] Makarova K.S. , Wolf Y.I., Iranzo J., Shmakov S.A., Alkhnbashi O.S., Brouns S.J.J., Charpentier E., Cheng D., Haft D.H., Horvath P. et al. Evolutionary classification of CRISPR–Cas systems: a burst of class 2 and derived variants. Nat. Rev. Microbiol. 2019; 18:67–83. 31857715 10.1038/s41579-019-0299-x PMC8905525

[3] Sternberg S.H. , Richter H., Charpentier E., Qimron U. Adaptation in CRISPR-Cas systems. Mol. Cell. 2016; 61:797–808. 26949040 10.1016/j.molcel.2016.01.030

[4] Mojica F.J.M. , Díez-Villaseñor C., García-Martínez J., Soria E. Intervening sequences of regularly spaced prokaryotic repeats derive from foreign genetic elements. J. Mol. Evol. 2005; 60:174–182. 15791728 10.1007/s00239-004-0046-3

[5] Charpentier E. , Richter H., van der Oost J., White M.F. Biogenesis pathways of RNA guides in archaeal and bacterial CRISPR-Cas adaptive immunity. FEMS Microbiol. Rev. 2015; 39:428–441. 25994611 10.1093/femsre/fuv023 PMC5965381

[6] Leenay R.T. , Beisel C.L. Deciphering, communicating, and engineering the CRISPR PAM. J. Mol. Biol. 2017; 429:177–191. 27916599 10.1016/j.jmb.2016.11.024 PMC5235977

[7] Marraffini L.A. , Sontheimer E.J. Self versus non-self discrimination during CRISPR RNA-directed immunity. Nature. 2010; 463:568–571. 20072129 10.1038/nature08703 PMC2813891

[8] Meeske A.J. , Marraffini L.A. RNA guide complementarity prevents self-targeting in type VI CRISPR systems. Mol. Cell. 2018; 71:791–801. 30122537 10.1016/j.molcel.2018.07.013 PMC7955661

[9] Deveau H. , Barrangou R., Garneau J.E., Labonté J., Fremaux C., Boyaval P., Romero D.A., Horvath P., Moineau S. Phage response to CRISPR-encoded resistance in *Streptococcus thermophilus*. J. Bacteriol. 2008; 190:1390–1400. 18065545 10.1128/JB.01412-07 PMC2238228

[10] Meeske A.J. , Nakandakari-Higa S., Marraffini L.A. Cas13-induced cellular dormancy prevents the rise of CRISPR-resistant bacteriophage. Nature. 2019; 570:241–245. 31142834 10.1038/s41586-019-1257-5 PMC6570424

[11] Abudayyeh O.O. , Gootenberg J.S., Konermann S., Joung J., Slaymaker I.M., Cox D.B.T., Shmakov S., Makarova K.S., Semenova E., Minakhin L. et al. C2c2 is a single-component programmable RNA-guided RNA-targeting CRISPR effector. Science. 2016; 353:aaf5573. 27256883 10.1126/science.aaf5573 PMC5127784

[12] Vercoe R.B. , Chang J.T., Dy R.L., Taylor C., Gristwood T., Clulow J.S., Richter C., Przybilski R., Pitman A.R., Fineran P.C. Cytotoxic chromosomal targeting by CRISPR/Cas systems can reshape bacterial genomes and expel or remodel pathogenicity islands. PLoS Genet. 2013; 9:e1003454. 23637624 10.1371/journal.pgen.1003454 PMC3630108

[13] Garneau J.E. , Dupuis M.-È., Villion M., Romero D.A., Barrangou R., Boyaval P., Fremaux C., Horvath P., Magadán A.H., Moineau S. The CRISPR/Cas bacterial immune system cleaves bacteriophage and plasmid DNA. Nature. 2010; 468:67–71. 21048762 10.1038/nature09523

[14] Dmytrenko O. , Neumann G.C., Hallmark T., Keiser D.J., Crowley V.M., Vialetto E., Mougiakos I., Wandera K.G., Domgaard H., Weber J. et al. Cas12a2 elicits abortive infection through RNA-triggered destruction of dsDNA. Nature. 2023; 613:588–594. 36599979 10.1038/s41586-022-05559-3 PMC9811890

[15] Stern A. , Keren L., Wurtzel O., Amitai G., Sorek R. Self-targeting by CRISPR: gene regulation or autoimmunity?. Trends Genet. 2010; 26:335–340. 20598393 10.1016/j.tig.2010.05.008 PMC2910793

[16] Nobrega F.L. , Walinga H., Dutilh B.E., Brouns S.J.J. Prophages are associated with extensive CRISPR–Cas auto-immunity. Nucleic Acids Res. 2020; 48:12074–12084. 33219687 10.1093/nar/gkaa1071 PMC7708048

[17] Gomaa A.A. , Klumpe H.E., Luo M.L., Selle K., Barrangou R., Beisel C.L. Programmable removal of bacterial strains by use of genome-targeting CRISPR-Cas systems. mBio. 2014; 5:e00928-13. 24473129 10.1128/mBio.00928-13 PMC3903277

[18] Wimmer F. , Beisel C.L. CRISPR-Cas systems and the paradox of self-targeting spacers. Front. Microbiol. 2019; 10:3078. 32038537 10.3389/fmicb.2019.03078 PMC6990116

[19] Zhang F. , Zhao S., Ren C., Zhu Y., Zhou H., Lai Y., Zhou F., Jia Y., Zheng K., Huang Z. CRISPRminer is a knowledge base for exploring CRISPR-Cas systems in microbe and phage interactions. Commun. Biol. 2018; 1:180. 30393777 10.1038/s42003-018-0184-6 PMC6208339

[20] Guan J. , Wang W., Sun B. Chromosomal targeting by the type III-A CRISPR-Cas system can reshape genomes in *Staphylococcus aureus*. mSphere. 2017; 2:e00403-17. 29152580 10.1128/mSphere.00403-17 PMC5687920

[21] Dy R.L. , Pitman A.R., Fineran P.C. Chromosomal targeting by CRISPR-Cas systems can contribute to genome plasticity in bacteria. Mob. Genet. Elements. 2013; 3:e26831. 24251073 10.4161/mge.26831 PMC3827097

[22] Selle K. , Klaenhammer T.R., Barrangou R. CRISPR-based screening of genomic island excision events in bacteria. Proc. Natl. Acad. Sci. U.S.A. 2015; 112:8076–8081. 26080436 10.1073/pnas.1508525112 PMC4491743

[23] Cañez C. , Selle K., Goh Y.J., Barrangou R. Outcomes and characterization of chromosomal self-targeting by native CRISPR-Cas systems in *Streptococcus thermophilus*. FEMS Microbiol. Lett. 2019; 366:fnz105. 31077282 10.1093/femsle/fnz105

[24] Watters K.E. , Fellmann C., Bai H.B., Ren S.M., Doudna J.A. Systematic discovery of natural CRISPR-Cas12a inhibitors. Science. 2018; 362:236–239. 30190307 10.1126/science.aau5138 PMC6185749

[25] Marino N.D. , Zhang J.Y., Borges A.L., Sousa A.A., Leon L.M., Rauch B.J., Walton R.T., Berry J.D., Joung J.K., Kleinstiver B.P. et al. Discovery of widespread type I and type V CRISPR-Cas inhibitors. Science. 2018; 362:240–242. 30190308 10.1126/science.aau5174 PMC6520112

[26] Li M. , Gong L., Cheng F., Yu H., Zhao D., Wang R., Wang T., Zhang S., Zhou J., Shmakov S.A. et al. Toxin-antitoxin RNA pairs safeguard CRISPR-Cas systems. Science. 2021; 372:eabe5601. 33926924 10.1126/science.abe5601

[27] Li R. , Fang L., Tan S., Yu M., Li X., He S., Wei Y., Li G., Jiang J., Wu M. Type I CRISPR-Cas targets endogenous genes and regulates virulence to evade mammalian host immunity. Cell Res. 2016; 26:1273–1287. 27857054 10.1038/cr.2016.135 PMC5143421

[28] Heussler G.E. , Cady K.C., Koeppen K., Bhuju S., Stanton B.A., O’Toole G.A Clustered regularly interspaced short palindromic repeat-dependent, biofilm-specific death of *Pseudomonas aeruginosa* mediated by increased expression of phage-related genes. mBio. 2015; 6:e00129–e15. 25968642 10.1128/mBio.00129-15 PMC4436051

[29] Ratner H.K. , Escalera-Maurer A., Le Rhun A., Jaggavarapu S., Wozniak J.E., Crispell E.K., Charpentier E., Weiss D.S. Catalytically active Cas9 mediates transcriptional interference to facilitate bacterial virulence. Mol. Cell. 2019; 75:498–510. 31256988 10.1016/j.molcel.2019.05.029 PMC7205310

[30] Cady K.C. , O’Toole G.A Non-identity-mediated CRISPR-bacteriophage interaction mediated via the Csy and Cas3 proteins. J. Bacteriol. 2011; 193:3433–3445. 21398535 10.1128/JB.01411-10 PMC3133329

[31] Zegans M.E. , Wagner J.C., Cady K.C., Murphy D.M., Hammond J.H., O’Toole G.A Interaction between bacteriophage DMS3 and host CRISPR region inhibits group behaviors of *Pseudomonas aeruginosa*. J. Bacteriol. 2009; 191:210–219. 18952788 10.1128/JB.00797-08 PMC2612449

[32] Wimmer F. , Mougiakos I., Englert F., Beisel C.L. Rapid cell-free characterization of multi-subunit CRISPR effectors and transposons. Mol. Cell. 2022; 82:1210–1224. 35216669 10.1016/j.molcel.2022.01.026

[33] Marshall R. , Maxwell C.S., Collins S.P., Jacobsen T., Luo M.L., Begemann M.B., Gray B.N., January E., Singer A., He Y. et al. Rapid and scalable characterization of CRISPR technologies using an *E. coli* cell-free transcription-translation system. Mol. Cell. 2018; 69:146–157. 29304331 10.1016/j.molcel.2017.12.007 PMC5976856

[34] Martin M. Cutadapt removes adapter sequences from high-throughput sequencing reads. EMBnet. journal. 2011; 17:10.

[35] Dobin A. , Davis C.A., Schlesinger F., Drenkow J., Zaleski C., Jha S., Batut P., Chaisson M., Gingeras T.R. STAR: ultrafast universal RNA-seq aligner. Bioinformatics. 2013; 29:15–21. 23104886 10.1093/bioinformatics/bts635 PMC3530905

[36] Anders S. , Pyl P.T., Huber W. HTSeq–a Python framework to work with high-throughput sequencing data. Bioinformatics. 2015; 31:166–169. 25260700 10.1093/bioinformatics/btu638 PMC4287950

[37] Langmead B. , Trapnell C., Pop M., Salzberg S.L. Ultrafast and memory-efficient alignment of short DNA sequences to the human genome. Genome Biol. 2009; 10:R25. 19261174 10.1186/gb-2009-10-3-r25 PMC2690996

[38] Langmead B. , Salzberg S.L. Fast gapped-read alignment with Bowtie 2. Nat. Methods. 2012; 9:357–359. 22388286 10.1038/nmeth.1923 PMC3322381

[39] Pawluk A. , Staals R.H.J., Taylor C., Watson B.N.J., Saha S., Fineran P.C., Maxwell K.L., Davidson A.R. Inactivation of CRISPR-Cas systems by anti-CRISPR proteins in diverse bacterial species. Nat. Microbiol. 2016; 1:16085. 27573108 10.1038/nmicrobiol.2016.85

[40] Pawluk A. , Bondy-Denomy J., Cheung V.H.W., Maxwell K.L., Davidson A.R. A new group of phage anti-CRISPR genes inhibits the type I-E CRISPR-Cas system of *Pseudomonas aeruginosa*. mBio. 2014; 5:e00896. 24736222 10.1128/mBio.00896-14 PMC3993853

[41] Yang H. , Patel D.J. Inhibition mechanism of an anti-CRISPR suppressor AcrIIA4 targeting SpyCas9. Mol. Cell. 2017; 67:117–127. 28602637 10.1016/j.molcel.2017.05.024 PMC5595222

[42] Berman H.M. , Westbrook J., Feng Z., Gilliland G., Bhat T.N., Weissig H., Shindyalov I.N., Bourne P.E. The Protein Data Bank. Nucleic Acids Res. 2000; 28:235–242. 10592235 10.1093/nar/28.1.235 PMC102472

[43] Pawluk A. , Amrani N., Zhang Y., Garcia B., Hidalgo-Reyes Y., Lee J., Edraki A., Shah M., Sontheimer E.J., Maxwell K.L. et al. Naturally occurring off-switches for CRISPR-Cas9. Cell. 2016; 167:1829–1838. 27984730 10.1016/j.cell.2016.11.017 PMC5757841

[44] Bondy-Denomy J. , Pawluk A., Maxwell K.L., Davidson A.R. Bacteriophage genes that inactivate the CRISPR/Cas bacterial immune system. Nature. 2013; 493:429–432. 23242138 10.1038/nature11723 PMC4931913

[45] Rauch B.J. , Silvis M.R., Hultquist J.F., Waters C.S., McGregor M.J., Krogan N.J., Bondy-Denomy J. Inhibition of CRISPR-Cas9 with bacteriophage proteins. Cell. 2017; 168:150–158. 28041849 10.1016/j.cell.2016.12.009 PMC5235966

[46] Hynes A.P. , Rousseau G.M., Lemay M.-L., Horvath P., Romero D.A., Fremaux C., Moineau S. An anti-CRISPR from a virulent streptococcal phage inhibits *Streptococcus pyogenes* Cas9. Nat. Microbiol. 2017; 2:1374–1380. 28785032 10.1038/s41564-017-0004-7

[47] Pawluk A. , Shah M., Mejdani M., Calmettes C., Moraes T.F., Davidson A.R., Maxwell K.L. Disabling a type I-E CRISPR-Cas nuclease with a bacteriophage-encoded anti-CRISPR protein. mBio. 2017; 8:e01751-17. 29233895 10.1128/mBio.01751-17 PMC5727412

[48] Pearson W.R. , Lipman D.J. Improved tools for biological sequence comparison. Proc. Natl. Acad. Sci. U.S.A. 1988; 85:2444–2448. 3162770 10.1073/pnas.85.8.2444 PMC280013

[49] Enright A.J. , Van Dongen S., Ouzounis C.A. An efficient algorithm for large-scale detection of protein families. Nucleic Acids Res. 2002; 30:1575–1584. 11917018 10.1093/nar/30.7.1575 PMC101833

[50] Alkhnbashi O.S. , Mitrofanov A., Bonidia R., Raden M., Tran V.D., Eggenhofer F., Shah S.A., Öztürk E., Padilha V.A., Sanches D.S. et al. CRISPRloci: comprehensive and accurate annotation of CRISPR-Cas systems. Nucleic Acids Res. 2021; 49:W125–W130. 34133710 10.1093/nar/gkab456 PMC8265192

[51] Edgar R.C. MUSCLE: multiple sequence alignment with high accuracy and high throughput. Nucleic Acids Res. 2004; 32:1792–1797. 15034147 10.1093/nar/gkh340 PMC390337

[52] Finn R.D. , Clements J., Eddy S.R. HMMER web server: interactive sequence similarity searching. Nucleic Acids Res. 2011; 39:W29–W37. 21593126 10.1093/nar/gkr367 PMC3125773

[53] Shin J. , Noireaux V. An *E. coli* cell-free expression toolbox: application to synthetic gene circuits and artificial cells. ACS Synth. Biol. 2012; 1:29–41. 23651008 10.1021/sb200016s

[54] Shin J. , Noireaux V. Efficient cell-free expression with the endogenous *E. coli* RNA polymerase and sigma factor 70. J. Biol. Eng. 2010; 4:8. 20576148 10.1186/1754-1611-4-8 PMC3161345

[55] Sievers F. , Wilm A., Dineen D., Gibson T.J., Karplus K., Li W., Lopez R., McWilliam H., Remmert M., Söding J. et al. Fast, scalable generation of high-quality protein multiple sequence alignments using Clustal Omega. Mol. Syst. Biol. 2011; 7:539. 21988835 10.1038/msb.2011.75 PMC3261699

[56] Mirdita M. , Schütze K., Moriwaki Y., Heo L., Ovchinnikov S., Steinegger M. ColabFold: making protein folding accessible to all. Nat. Methods. 2022; 19:679–682. 35637307 10.1038/s41592-022-01488-1 PMC9184281

[57] Jumper J. , Evans R., Pritzel A., Green T., Figurnov M., Ronneberger O., Tunyasuvunakool K., Bates R., Žídek A., Potapenko A. et al. Highly accurate protein structure prediction with AlphaFold. Nature. 2021; 596:583–589. 34265844 10.1038/s41586-021-03819-2 PMC8371605

[58] Sehnal D. , Bittrich S., Deshpande M., Svobodová R., Berka K., Bazgier V., Velankar S., Burley S.K., Koča J., Rose A.S. Mol* Viewer: modern web app for 3D visualization and analysis of large biomolecular structures. Nucleic Acids Res. 2021; 49:W431–W437. 33956157 10.1093/nar/gkab314 PMC8262734

[59] Zhang Y. , Skolnick J. TM-align: a protein structure alignment algorithm based on the TM-score. Nucleic Acids Res. 2005; 33:2302–2309. 15849316 10.1093/nar/gki524 PMC1084323

[60] Edgar R.C. MUSCLE: a multiple sequence alignment method with reduced time and space complexity. BMC Bioinf. 2004; 5:113. 10.1186/1471-2105-5-113 PMC517706 15318951

[61] Jore M.M. , Lundgren M., van Duijn E., Bultema J.B., Westra E.R., Waghmare S.P., Wiedenheft B., Pul U., Wurm R., Wagner R. et al. Structural basis for CRISPR RNA-guided DNA recognition by Cascade. Nat. Struct. Mol. Biol. 2011; 18:529–536. 21460843 10.1038/nsmb.2019

[62] Hochstrasser M.L. , Taylor D.W., Bhat P., Guegler C.K., Sternberg S.H., Nogales E., Doudna J.A. CasA mediates Cas3-catalyzed target degradation during CRISPR RNA-guided interference. Proc. Natl. Acad. Sci. U.S.A. 2014; 111:6618–6623. 24748111 10.1073/pnas.1405079111 PMC4020112

[63] Westra E.R. , van Erp P.B.G., Künne T., Wong S.P., Staals R.H.J., Seegers C.L.C., Bollen S., Jore M.M., Semenova E., Severinov K. et al. CRISPR immunity relies on the consecutive binding and degradation of negatively supercoiled invader DNA by Cascade and Cas3. Mol. Cell. 2012; 46:595–605. 22521689 10.1016/j.molcel.2012.03.018 PMC3372689

[64] Huo Y. , Nam K.H., Ding F., Lee H., Wu L., Xiao Y., Farchione M.D. Jr, Zhou S., Rajashankar K., Kurinov I. et al. Structures of CRISPR Cas3 offer mechanistic insights into Cascade-activated DNA unwinding and degradation. Nat. Struct. Mol. Biol. 2014; 21:771–777. 25132177 10.1038/nsmb.2875 PMC4156918

[65] Xiao Y. , Luo M., Dolan A.E., Liao M., Ke A. Structure basis for RNA-guided DNA degradation by Cascade and Cas3. Science. 2018; 361:eaat0839. 29880725 10.1126/science.aat0839 PMC6537108

[66] Morinière L. , Lecomte S., Gueguen E., Bertolla F. In vitro exploration of the *Xanthomonas hortorum* pv. *vitians* genome using transposon insertion sequencing and comparative genomics to discriminate between core and contextual essential genes. Microbial Genomics. 2019; 7:000546. 33760724 10.1099/mgen.0.000546 PMC8627662

[67] Brouns S.J.J. , Jore M.M., Lundgren M., Westra E.R., Slijkhuis R.J.H., Snijders A.P.L., Dickman M.J., Makarova K.S., Koonin E.V., van der Oost J. Small CRISPR RNAs guide antiviral defense in prokaryotes. Science. 2008; 321:960–964. 18703739 10.1126/science.1159689 PMC5898235

[68] Haurwitz R.E. , Jinek M., Wiedenheft B., Zhou K., Doudna J.A. Sequence- and structure-specific RNA processing by a CRISPR endonuclease. Science. 2010; 329:1355–1358. 20829488 10.1126/science.1192272 PMC3133607

[69] Luo M.L. , Mullis A.S., Leenay R.T., Beisel C.L. Repurposing endogenous type I CRISPR-Cas systems for programmable gene repression. Nucleic Acids Res. 2015; 43:674–681. 25326321 10.1093/nar/gku971 PMC4288209

[70] Rath D. , Amlinger L., Hoekzema M., Devulapally P.R., Lundgren M. Efficient programmable gene silencing by Cascade. Nucleic Acids Res. 2015; 43:237–246. 25435544 10.1093/nar/gku1257 PMC4288158

[71] Davidson A.R. , Lu W.-T., Stanley S.Y., Wang J., Mejdani M., Trost C.N., Hicks B.T., Lee J., Sontheimer E.J. Anti-CRISPRs: protein inhibitors of CRISPR-Cas systems. Annu. Rev. Biochem. 2020; 89:309–332. 32186918 10.1146/annurev-biochem-011420-111224 PMC9718424

[72] Gussow A.B. , Park A.E., Borges A.L., Shmakov S.A., Makarova K.S., Wolf Y.I., Bondy-Denomy J., Koonin E.V. Machine-learning approach expands the repertoire of anti-CRISPR protein families. Nat. Commun. 2020; 11:3784. 32728052 10.1038/s41467-020-17652-0 PMC7391736

[73] Roux S. , Enault F., Hurwitz B.L., Sullivan M.B. VirSorter: mining viral signal from microbial genomic data. PeerJ. 2015; 3:e985. 26038737 10.7717/peerj.985 PMC4451026

[74] Song W. , Sun H.-X., Zhang C., Cheng L., Peng Y., Deng Z., Wang D., Wang Y., Hu M., Liu W. et al. Prophage Hunter: an integrative hunting tool for active prophages. Nucleic Acids Res. 2019; 47:W74–W80. 31114893 10.1093/nar/gkz380 PMC6602508

[75] Arndt D. , Grant J.R., Marcu A., Sajed T., Pon A., Liang Y., Wishart D.S. PHASTER: a better, faster version of the PHAST phage search tool. Nucleic Acids Res. 2016; 44:W16–W21. 27141966 10.1093/nar/gkw387 PMC4987931

[76] Zhou Y. , Liang Y., Lynch K.H., Dennis J.J., Wishart D.S. PHAST: a fast phage search tool. Nucleic Acids Res. 2011; 39:W347–W52. 21672955 10.1093/nar/gkr485 PMC3125810

[77] Wandera K.G. , Collins S.P., Wimmer F., Marshall R., Noireaux V., Beisel C.L. An enhanced assay to characterize anti-CRISPR proteins using a cell-free transcription-translation system. Methods. 2020; 172:42–50. 31121300 10.1016/j.ymeth.2019.05.014

[78] Bondy-Denomy J. , Davidson A.R., Doudna J.A., Fineran P.C., Maxwell K.L., Moineau S., Peng X., Sontheimer E.J., Wiedenheft B. A Unified Resource for Tracking Anti-CRISPR Names. CRISPR J. 2018; 1:304–305. 31021273 10.1089/crispr.2018.0043 PMC10625466

[79] Serfiotis-Mitsa D. , Herbert A.P., Roberts G.A., Soares D.C., White J.H., Blakely G.W., Uhrín D., Dryden D.T.F. The structure of the KlcA and ArdB proteins reveals a novel fold and antirestriction activity against Type I DNA restriction systems in vivo but not in vitro. Nucleic Acids Res. 2010; 38:1723–1737. 20007596 10.1093/nar/gkp1144 PMC2836571

[80] Niu Y. , Yang L., Gao T., Dong C., Zhang B., Yin P., Hopp A.-K., Li D., Gan R., Wang H. et al. A type I-F anti-CRISPR protein inhibits the CRISPR-Cas surveillance complex by ADP-ribosylation. Mol. Cell. 2020; 80:512–524. 33049228 10.1016/j.molcel.2020.09.015

[81] Knott G.J. , Thornton B.W., Lobba M.J., Liu J.-J., Al-Shayeb B., Watters K.E., Doudna J.A. Broad-spectrum enzymatic inhibition of CRISPR-Cas12a. Nat. Struct. Mol. Biol. 2019; 26:315–321. 30936531 10.1038/s41594-019-0208-z PMC6449189

[82] Dong L. , Guan X., Li N., Zhang F., Zhu Y., Ren K., Yu L., Zhou F., Han Z., Gao N. et al. An anti-CRISPR protein disables type V Cas12a by acetylation. Nat. Struct. Mol. Biol. 2019; 26:308–314. 30936526 10.1038/s41594-019-0206-1

[83] Wang J. , Ma J., Cheng Z., Meng X., You L., Wang M., Zhang X., Wang Y. A CRISPR evolutionary arms race: structural insights into viral anti-CRISPR/Cas responses. Cell Res. 2016; 26:1165–1168. 27585537 10.1038/cr.2016.103 PMC5113301

[84] Wang X. , Yao D., Xu J.-G., Li A.-R., Xu J., Fu P., Zhou Y., Zhu Y. Structural basis of Cas3 inhibition by the bacteriophage protein AcrF3. Nat. Struct. Mol. Biol. 2016; 23:868–870. 27455460 10.1038/nsmb.3269

[85] Bondy-Denomy J. , Garcia B., Strum S., Du M., Rollins M.F., Hidalgo-Reyes Y., Wiedenheft B., Maxwell K.L., Davidson A.R. Multiple mechanisms for CRISPR-Cas inhibition by anti-CRISPR proteins. Nature. 2015; 526:136–139. 26416740 10.1038/nature15254 PMC4935067

[86] Pinilla-Redondo R. , Shehreen S., Marino N.D., Fagerlund R.D., Brown C.M., Sørensen S.J., Fineran P.C., Bondy-Denomy J. Discovery of multiple anti-CRISPRs highlights anti-defense gene clustering in mobile genetic elements. Nat. Commun. 2020; 11:5652. 33159058 10.1038/s41467-020-19415-3 PMC7648647

[87] León L.M. , Park A.E., Borges A.L., Zhang J.Y., Bondy-Denomy J. Mobile element warfare via CRISPR and anti-CRISPR in *Pseudomonas aeruginosa*. Nucleic Acids Res. 2021; 49:2114–2125. 33544853 10.1093/nar/gkab006 PMC7913775

[88] Mejdani M. , Pawluk A., Maxwell K.L., Davidson A.R. Anti-CRISPR AcrIE2 binds the type I-E CRISPR-Cas complex but does not block DNA binding. J. Mol. Biol. 2021; 433:166759. 33338493 10.1016/j.jmb.2020.166759

[89] Xie Y. , Zhang L., Gao Z., Yin P., Wang H., Li H., Chen Z., Zhang Y., Yang M., Feng Y. AcrIF5 specifically targets DNA-bound CRISPR-Cas surveillance complex for inhibition. Nat. Chem. Biol. 2022; 18:670–677. 35301482 10.1038/s41589-022-00995-8

[90] Isaev A. , Drobiazko A., Sierro N., Gordeeva J., Yosef I., Qimron U., Ivanov N.V., Severinov K. Phage T7 DNA mimic protein Ocr is a potent inhibitor of BREX defence. Nucleic Acids Res. 2020; 48:5397–5406. 32338761 10.1093/nar/gkaa290 PMC7261183

[91] Kennaway C.K. , Obarska-Kosinska A., White J.H., Tuszynska I., Cooper L.P., Bujnicki J.M., Trinick J., Dryden D.T.F. The structure of M.EcoKI Type I DNA methyltransferase with a DNA mimic antirestriction protein. Nucleic Acids Res. 2009; 37:762–770. 19074193 10.1093/nar/gkn988 PMC2647291

[92] Ho P. , Chen Y., Biswas S., Canfield E., Feldman D.E. Bacteriophage anti-defense genes that neutralize TIR and STING immune responses. Cell Rep. 2022; 42:112305. 10.1016/j.celrep.2023.112305 36952342

[93] Leavitt A. , Yirmiya E., Amitai G., Lu A., Garb J., Herbst E., Morehouse B.R., Hobbs S.J., Antine S.P., Sun Z.-Y.J. et al. Viruses inhibit TIR gcADPR signalling to overcome bacterial defence. Nature. 2022; 611:326–331. 36174646 10.1038/s41586-022-05375-9

[94] Yirmiya E. , Leavitt A., Lu A., Avraham C., Osterman I., Garb J., Antine S.P., Mooney S.E., Hobbs S.J., Kranzusch P.J. et al. Phages overcome bacterial immunity via diverse anti-defense proteins. 2023; bioRxiv doi:01 May 2023, preprint: not peer reviewed 10.1101/2023.05.01.538930. 37992756

[95] Hobbs S.J. , Wein T., Lu A., Morehouse B.R., Schnabel J., Leavitt A., Yirmiya E., Sorek R., Kranzusch P.J. Phage anti-CBASS and anti-Pycsar nucleases subvert bacterial immunity. Nature. 2022; 605:522–526. 35395152 10.1038/s41586-022-04716-y PMC9117128

[96] Barrangou R. , Fremaux C., Deveau H., Richards M., Boyaval P., Moineau S., Romero D.A., Horvath P. CRISPR provides acquired resistance against viruses in prokaryotes. Science. 2007; 315:1709–1712. 17379808 10.1126/science.1138140

