# Supplementary material for: Interrogating two extensively self-targeting Type I CRISPR-Cas systems in Xanthomonas albilineans reveals distinct anti-CRISPR proteins that block DNA degradation
Source: Nucleic Acids Res. 2023 Nov 28;52(2):769–83. doi: 10.1093/nar/gkad1097 (PMC10810201; doi:10.1093/nar/gkad1097)
Supplement: gkad1097_Supplemental_Files [file gkad1097_supplemental_files.zip › Xal self-targeting SI.pdf]

SUPPLEMENTARY INFORMATION

Interrogating two extensively self-targeting Type I CRISPR-Cas systems in *Xanthomonas albilineans* reveals distinct anti-CRISPR proteins that block DNA degradation

Franziska Wimmer, Frank Englert*, Katharina G. Wandera*, Omer S. Alkhnbashi, Scott P. Collins, Rolf Backofen, Chase L. Beisel[@]

*Equal contributions

[@]Correspondence: chase.beisel@helmholtz-hiri.de (to C.L.B.)

SUPPLEMENTARY FIGURES

A

gene ID	Name	TPM
XALC_2885	IC *cas3*	31
XALC_2887	IC *cas5*	910
XALC_2888	IC *cas8*	94
XALC_2889	IC *cas7*	146
XALC_2890	IC *cas4*	33
XALC_2891	IC *cas1*	27
XALC_2892	IC *cas2*	89

gene ID	Name	TPM
XALC_3048	IF *cas1*	10
XALC_3049	IF *cas2-3*	7
XALC_3050	IF *cas8*	324
XALC_3051	IF *cas5*	115
XALC_3052	IF *cas7*	87
XALC_3053	IF *cas6*	118

gene ID	Name	TPM
XALC_0251	*birA*	93
XALC_0807	*mreD*	44
XALC_0808	*mrdA*	86
XALC_1005	*nuoG*	964
XALC_1665	*purB*	55
XALC_2038	*lpxB*	116
XALC_2040	*dnaE*	149
XALC_2768	*thyA*	326
XALC_2997	*hemB*	130
XALC_3172	*rnpA*	77

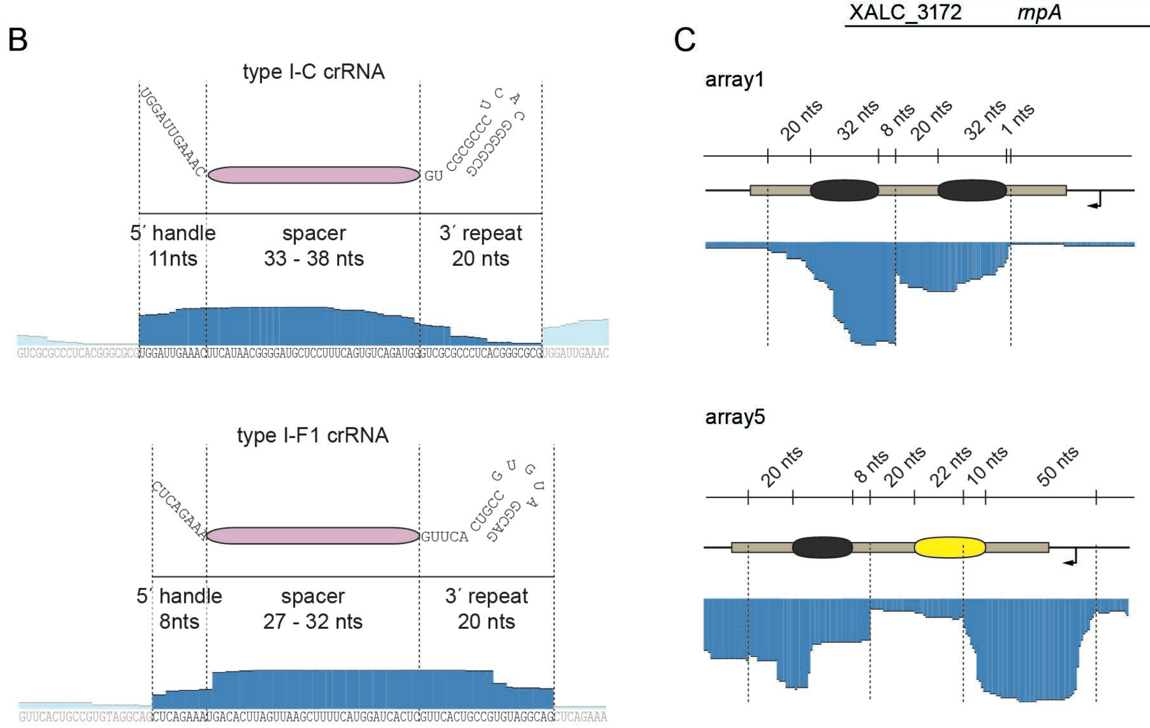

Figure S1: RNA-Seq reveals transcription of *X. albilineans cas* genes and crRNA processing. (**A**) Transcripts per million calculated from total RNA-Seq results. TPM of type I-C and type I-F1 *cas* genes are shown. Right table: essential genes shown for comparison. (**B**) Representative crRNAs undergoing standard processing. Expected mature crRNAs for the *X. albilineans* type I-C and type I-F1 arrays are shown. (**C**) Atypical processing for crRNAs derived from CRISPR array 1 and array 5. Small RNA-Seq coverage of type I-F1 array 1 and array 5 are shown in blue. Self-targeting spacers are depicted in yellow ovals and non self-targeting spacers are represented in black ovals. Dashed lines display the location of the processing event.

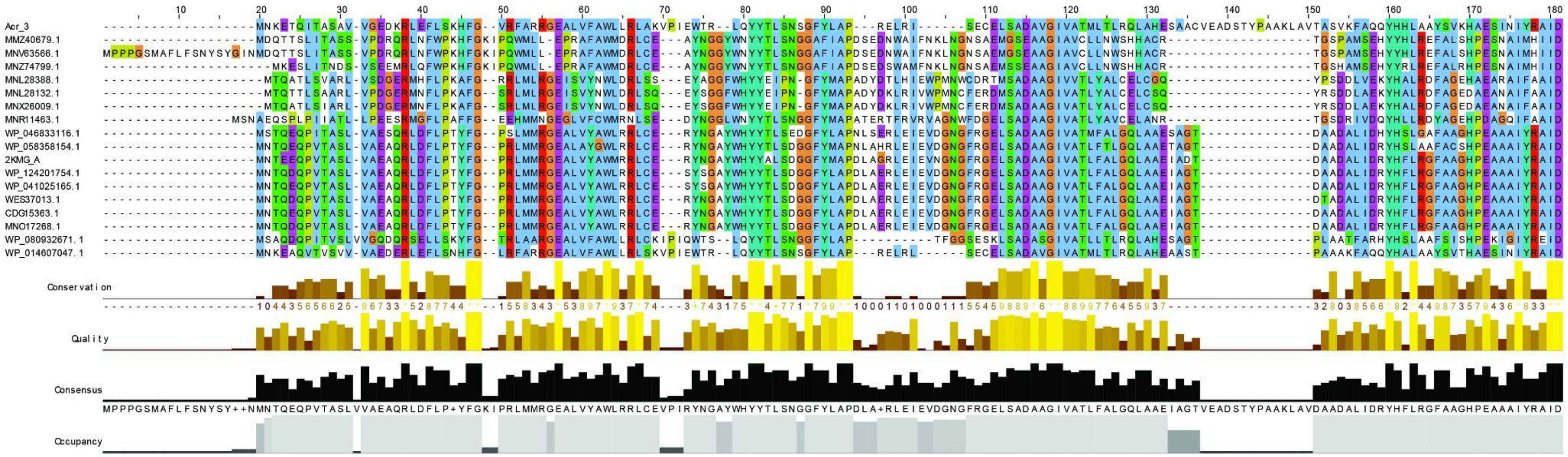

Figure S2: Amino-acid conservation of identified Acr_3 homolog.

A

```
KlcA            MNTEEQPVTASLVAEAQRLDFLPTYFGPRLMMRGEALVYAWMRRLCE-RYNGAYWHYYAL
AcrIC11         MNKETQITASAVVGEDKRLEFLSKHFGVRFARRGEALVFAWLLRLAKVPIEWTRLQYYTL
                **.* * .::::*.* :**:**..:** *:  ******:**: **.:   : :  :**:*

KlcA            SDGGFYMAPDLAGRLEIEVNGNGFRGELSADAAGIVATLFALGQLAAEIADTDAADA---
AcrIC11         SNSGFYLAPR-----ELRIS----ECELSADAVGIVATMLTLRQLAHESAACVEADSTYP
                *:.***:**      *: :.      ******.*****:::* * *        **:

KlcA            -----------LIDRYHFLRGFAAGHPEAAAIYRAID
AcrIC11         AAKLAVTASVKFAQQYHHLAAYSVKHAESINIYRAID
                           : :.**.* .::. *.*:  ******
```

B

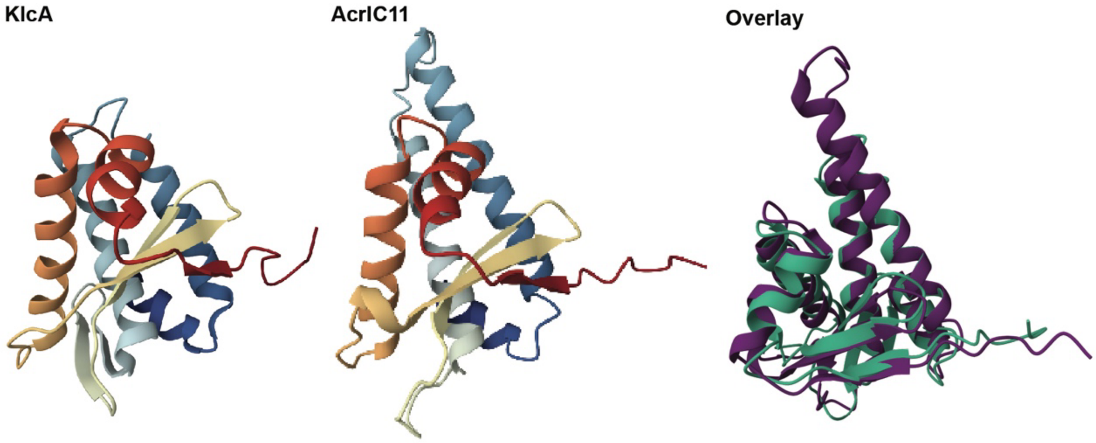

Figure S3. Comparison of the predicted structure of AcrIC11 with the crystal structure of KlcA. (**A**) Amino acid sequence alignment between KlcA and AcrIC11 using MUSCLE. (**B**) Structures of KlcA and AcrIC11. The published structure of KlcA is derived from crystallography (2KMG) (1), while the structure of AcrIC11 was predicted using AlphaFold (2, 3). The overlaid structure was generated using TM-align (4) and indicated strong similarity (RMSD = 1.467 Å, TM = 0.79111).

SUPPLEMENTARY TABLES

Table S1: List of plasmids used in the work. See the attached Excel file.

Table S2: Genomic location of predicted prophage regions. Prophage regions are predicted with VirSorter v1.0.3 (5), Prophage Hunter (6) and PHASTER (7, 8).

Used tool	Genomic region	Comment
VirSorter	204,842-292,722	prophage category 5
Prophage Hunter	217,510-251,216	active
PHASTER	251,524-271,683	incomplete
VirSorter	1,197,275-1,218,686	prophage category 6
Prophage Hunter	1,691,999-1,713,262	ambiguous
VirSorter	1,696,720-1,861,289	prophage category 5
PHASTER	1,790,323-1,798,253	intact
Prophage Hunter	2,174,815-2,185,313	ambiguous
Prophage Hunter	2,830,643-2,860,815	ambiguous
PHASTER	2,837,733-2,874,463	questionable
PHASTER	2,841,444-2,856,796	incomplete
PHASTER	3,098,545-3,132,674	questionable
Prophage Hunter	3,102,291-3,128,520	ambiguous
PHASTER	3,107,687-3,129,023	incomplete
Prophage Hunter	3,120,278-3,132,701	ambiguous

Table S3: Genomic location of putative Acrs and their amino acid sequence. TPM: transcripts per million based on RNA-seq analyses.

Acr cand.	Genomic location	TPM	AA sequence	Comment
Acr_1	265340-265717	316	MNYMKKWIREHVAEVIKANELSRWVDDS DMKFAMYVVECGQGAQLAQDVGREIGN ETIVAIAQTVIDTIDEVSRGGTPRTRSRRKI TDKQRHVLAVVLLEKYGTARGIAAAGWG LTDEEIDNADV*	Identified by HMM profile search
Acr_2	2852041-2852406;3116105-3116470	0; 0	MPRKAPTPCRHPGCGKLVSDGSGYCAD HQRDKVGWHKDRRNAHQRGYGATWQK LRAFVMQRDQGLCQPCKQSGRLTPAVA VDHIVPKSQGGTDHPNNCQAICHRCHVL KTAQESHQGREGA*	Identified by HMM profile search
Acr_3	plasmII: 16827-17273	55	MNKETQITASAVVGEDKRLEFLSKHFGV RFARRGEALVFAWLLRLAKVPIEWTRLQ YYTLSNSGFYLAPRELRISECELSADAVGI VATMLTLRQLAHESAACVEADSTYPAAK LAVTASVKFAQQYHHLAAYSVKHAESINI YRAID*	Identified by guilt-by-association
Acr_4	plasmIII: 7731-7997	0	MSTLTVTTRGQVTFRAEVLQHLGIKPGEK IEVYLMPDGRAELKAAKPKGSFRELRGIL KHKTNGARLSIEEINDAIAEAGDAAGTGN T*	Identified by HMM profile search
Acr_5	2852137-2852406; 3116201-3116470	0; 0	MVGWHKDRRNAHQRGYGATWQKLRAF VMQRDQGLCQPCKQSGRLTPAVAVDHI VPKSQGGTDHPNNCQAICHRCHVLKTAQ ESHQGREGA*	Identified by HMM profile search, N-terminal M added; part of Acr2
Acr_6	2852659-2852865	4	MQSQKTARPLNFSRVQNEKLFLDRTVSE LSVARDYKADLAQIEQIDATPWTAASHAD MTSELKTYARS*	Identified by HMM profile search
Acr_7	2852734-2852865	0	MVSELSVARDYKADLAQIEQIDATPWTAA SHADMTSELKTYARS*	Identified by guilt-by-association, N-terminal M added; part of Acr6

Acr_8	plasmIII: 8405-8809	45	MNKEAQVTVSVVVAEDERLEFLSNHFGLRFARRGEALVFAWLLRLSKVPIEWTRLQYYTLSNSGFYLAPRELRLSECELSADAVGIVATMLTLRQLAHEAASTPAAAKFAQQYHALAAYSVTHAESINIYRAID*	Identified by HMM profile search
Acr_9	plasmIII: 8893-9117	0	MRVFNIAEIEFAINYWRTRIVPDDGALMCAPALSLLQLYGHMIFDRIEAVPESELDAEQGVALSVALYQHELPL*	Identified by HMM profile search
Acr_10	3116786-3116941	188	LEKSVSELSAARDYKADLAQIGQIDVTPWTAAAHADMTPAEPVELEPYARS*	Identified by guilt-by-association
Acr_11	2852137-2852406; 3116201-3116470	0; 0	VGWHKDRRNAHQRGYGATWQKLRAFVMQRDQGLCQPCKQSGRLTPAVAVDHIVPKSQGGTDHPNNCQAICHRCHVLKTAQESHQGREGA*	Identified by HMM profile search, Acr5 without N-terminal M; part of Acr2
Acr_12	2845745-2845960	0	MVDAKHAAAALRLPYYWFSDQAMRNKYRIPHYLLGGLVRYRLSELSAWAARSTLVQRSDTSNVGTSTEEAE*	Identified by HMM profile search
Acr_13	2845431-2845706	1	MNLITSLRHKLSYLYGEHLPNEIHYHRADGQHVVVALQDATVDQLAFAIQTINTESVALSRHRNALEELHTEVRKRSACGADRIADVAWDN*	Identified by HMM profile search
Acr_14	2844724-2844861	2	MADGSAPLPSLTTLPPRDHAMRSLDEFVRVDDGRNHKPAHKSRHT*	Identified by guilt-by-association
Acr_15	1430699-1431022; 1698203-1698526; 1917087-1917410; 2197118-2197441; 2828433-2828756; 3118350-3118673	0; 2; 0; 6; 2; 3	MSKSNKFSPEVRERAVRMVQEQRGEYQSLWAAIESIAPKIGCVPQTLNEWVKRAEVDAGAREGVTSSEAQRMKELEREVKELRRANEILKLASAFFAQAELDRRLKS*	Identified by HMM profile search
Acr_16	2845054-2845317	1	MTTRLPATQIGQLCESKDPGSTTRIALDESELAARWGLSVKTLRRWRQEQLGPVFCKLGARVTYLICDVEAFEQRVSRYSTFARAYP*	Identified by HMM profile search
Acr_17	2773986-2774279; 3111383-3111676	0; 0	MQRITRRRYTDDFKAQAIALAESVGLAKAARQLGMSVKTLANWLGASRGGQPLSSPSRKPVSEMESELARLRAENATLKMEREILKKATAFFARESK*	Identified by HMM profile search

Table S4: Features of the self-targeting spacers. See the attached Excel file.

Table S5: Mapped read counts for the two biological replicates for the expressed crRNAs. See the attached Excel file.

Table S6: Average mapped read counts for all genes in the major predicted prophage regions. See the attached Excel file.

SUPPLEMENTARY REFERENCES

1. Berman,H.M., Westbrook,J., Feng,Z., Gilliland,G., Bhat,T.N., Weissig,H., Shindyalov,I.N. and Bourne,P.E. (2000) The Protein Data Bank. *Nucleic Acids Res.*, **28**, 235–242.

2. Mirdita,M., Schütze,K., Moriwaki,Y., Heo,L., Ovchinnikov,S. and Steinegger,M. (2022) ColabFold: making protein folding accessible to all. *Nat. Methods*, **19**, 679–682.

3. Jumper,J., Evans,R., Pritzel,A., Green,T., Figurnov,M., Ronneberger,O., Tunyasuvunakool,K., Bates,R., Žídek,A., Potapenko,A., *et al.* (2021) Highly accurate protein structure prediction with AlphaFold. *Nature*, **596**, 583–589.

4. Zhang,Y. and Skolnick,J. (2005) TM-align: a protein structure alignment algorithm based on the TM-score. *Nucleic Acids Res.*, **33**, 2302–2309.

5. Roux,S., Enault,F., Hurwitz,B.L. and Sullivan,M.B. (2015) VirSorter: mining viral signal from microbial genomic data. *PeerJ*, **3**, e985.

6. Song,W., Sun,H.-X., Zhang,C., Cheng,L., Peng,Y., Deng,Z., Wang,D., Wang,Y., Hu,M., Liu,W., *et al.* (2019) Prophage Hunter: an integrative hunting tool for active prophages. *Nucleic Acids Res.*, **47**, W74–W80.

7. Arndt,D., Grant,J.R., Marcu,A., Sajed,T., Pon,A., Liang,Y. and Wishart,D.S. (2016) PHASTER: a better, faster version of the PHAST phage search tool. *Nucleic Acids Res.*, **44**, W16–21.

8. Zhou,Y., Liang,Y., Lynch,K.H., Dennis,J.J. and Wishart,D.S. (2011) PHAST: a fast phage search tool. *Nucleic Acids Res.*, **39**, W347–52.
